# Indisulam synergizes with melphalan to inhibit Multiple Myeloma malignancy via targeting TOP2A

**DOI:** 10.1371/journal.pone.0299019

**Published:** 2024-04-09

**Authors:** Chengyu Wu, Chao Wu, Jia Liu, Mingyuan Jia, Xinyi Zeng, Ze Fu, Ziqi He, Wenbin Xu, Hua Yan

**Affiliations:** 1 Shanghai Institute of Hematology, State Key Laboratory of Medical Genomics, National Research Center for Translational Medicine at Shanghai, Ruijin Hospital, Shanghai Jiao Tong University School of Medicine, Shanghai, China; 2 Department of General Practice, Ruijin Hospital, Shanghai Jiao Tong University School of Medicine, Shanghai, China; 3 Department of Hematology, Huadong Hospital Affiliated with Fudan University, Shanghai, China; BRAC University, BANGLADESH

## Abstract

Multiple myeloma (MM) is the second most prevalent hematologic malignancy which remains uncurable. Numerous drugs have been discovered to inhibit MM cells. Indisulam, an aryl sulfonamide, has a potent anti-myeloma activity in vitro and in vivo. This study aims to explore the new mechanism of indisulam and investigate its potential use in combination with melphalan. We examined DNA damage in MM cells through various methods such as western blotting (WB), immunofluorescence, and comet assay. We also identified the role of topoisomerase IIα (TOP2A) using bioinformatic analyses. The impact of indisulam on the RNA and protein levels of TOP2A was investigated through qPCR and WB. Cell proliferation and apoptosis were assessed using CCK-8 assays, Annexin V/PI assays and WB. We predicted the synergistic effect of the combination treatment based on calculations performed on a website, and further explored the effect of indisulam in combination with melphalan on MM cell lines and xenografts. RNA sequencing data and basic experiments indicated that indisulam caused DNA damage and inhibited TOP2A expression by decreasing transcription and promoting degradation via the proteasome pathway. Functional experiments revealed that silencing TOP2A inhibited cell proliferation and induced apoptosis and DNA damage. Finally, Indisulam/melphalan combination treatment demonstrated a strong synergistic anti-tumor effect compared to single-agent treatments in vitro and in vivo. These findings suggest that combination therapies incorporating indisulam and melphalan have the potential to enhance treatment outcomes for MM.

## Introduction

Multiple Myeloma (MM) is a malignancy characterized by abnormal proliferation of plasma cells. The introduction of novel agents has significantly prolonged the lifespan of patients with MM, with a 5-year survival rate of 50% [1, 2]. However, most of the patients eventually develop resistance to treatment and experience relapse [3]. Therefore, it is necessary to explore novel therapeutic approaches.

Indisulam, a novel sulfonamide anticancer agent, is currently being studied for its its mechanism in cancer treatment. Indisulam was first identified as a cell cycle blocker, inhibiting the activation of CDK2 and Cyclin E, decreasing the expression of Cyclin A, Cyclin B1 and CDK2 and arresting tumor cells in G1 phase [4, 5]. Further research has indicated that indisulam also inhibits the activity of carbonic anhydrase IX, which plays a role in acidifying the tumor environment and promoting tumor metastasis through the efficient catalysis of carbon dioxide hydration to generate bicarbonate and protons [6, 7]. Recently, indisulam has been proved as a natural molecular glue that binds to RNA binding motif protein 39 (RBM39) and regulates alternative splicing to exert anti-tumor effects [8, 9]. However, the mechanism of indisulam awaits further exploration.

Indisulam has shown promising results in preclinical studies for the treatment of various types of cancer, including leukemia [10], colon cancer [11] and gastric cancer [12]. Studies have shown that indisulam has a good safety profile and is effective in combination with other therapies [13, 14]. Indisulam was reported to have synergistic effects in combination with a variety of common antineoplastic agents, such as 5-fluorouracil, paclitaxel, gemcitabine [15] and palbociclib [16]. In addition, a phase II clinical trial of indisulam combined with idarubicin and cytarabine in the treatment of relapsed/refractory acute myeloid leukemia (AML) showed that although indisulam alone had no significant therapeutic effect, 35% patients benefited from combination therapy, with an estimated 1-year survival rate of 51% [17]. This provides a theoretical and practical basis for the use of indisulam in combination with chemotherapeutic agents in other hematological neoplasms.

Melphalan is a commonly used alkylating agent in the treatment of MM. It binds to the N7 of the guanine base of DNA, resulting in DNA double-strand cross-linking [18]. This leads to the inhibition of DNA replication and an increase in the topological pressure of DNA in MM cells. As a result, cell cycle arrest and cell death occur [19]. Resistance to melphalan can occur due to the activation of DNA repair pathways [20].

Currently, the potential application value and mechanism of indisulam in MM have not been studied. Previous studies of our group have shown that indisulam exerts great anti-tumor effects in MM in vitro and in vivo. Thus, we aim to further investigate the mechanism of indisulam on MM and explore a combination regimen containing indisulam and clinical chemotherapeutic drugs.

## Materials and methods

### Cell culture and reagent

NCI-H929, MM.1S, RPMI-8226, U266, OPM2 and HEK293T cells were obtained from the Cell Resource Center of Shanghai Institute for Biological Science. NCI-H929 MM.1S and U266 cells were cultured in RPMI-1640 (BasalMedia, Shanghai, China) containing 10% fetal bovine serum (FBS) and 100IU/ml penicillin and 100lg/ml streptomycin. RPMI-8226 was cultured in RPMI-1640 with 20% FBS. OPM2 was cultured in IMDM (BasalMedia, Shanghai, China) with 20% FBS. HEK293T was cultured in DMDM (BasalMedia, Shanghai, China) with 10% FBS. Indisulam was purchased from MedChem Express, and was suspended in dimethyl sulfoxide (DMSO). The stock concentration of indisulam was 40mM. Melphalan was purchased from Sigma-Aldrich. The stock concentration of melphalan was 40mM. The final DMSO concentration in each sample is below 0.1%.

### Plasmid construction and transfection

A short hairpin RNA (shRNA) sequence against TOP2A or a scramble sequence was inserted into the pLKO.1 vector. The sequences are listed in S2 Table in S1 File. To establish a cell line expressing shRNA against TOP2A, lentiviral particles were produced in HEK293T cells by co-transfection of psPAX2 and pMD2.G plasmid, using polyethylenimine (PEI). Cells were infected in culture medium in the presence of polybrene (8 μg/mL) for 6 hours.

### Western blotting assay

Whole cell lysates prepared using 2×SDS were separated by SDS-polyacrylamide gel, and then transferred onto nitrocellulose membranes (Bio-Rad, Hercules, CA). Membranes were blocked for 30 minutes with 5% nonfat milk and incubated with primary antibodies overnight at 4°C, followed by incubation of HRP-linked secondary antibodies (Cell Signaling Technology, Danvers, MA) for 1 hour at room temperature and detection using chemiluminescence phototope-HRP kit (Cell Signaling Technology, Danvers, MA). The primary antibodies are listed in S1 Table in S1 File.

### Immunofluorescence

A total of 1×10^5^ cells were resuspended to 1mL with PBS. Cells were centrifuged onto glass slides using Cytocentrifuge (Thermo Fisher Scientific, Waltham, MA) at 800rpm for 5 minutes, and then fixed with 4% Paraformaldehyde (PFA) for 15 minutes. Samples were incubated with 0.5% Triton X-100 for 15 minutes at room temperature and blocked with 2% bovine serum albumin (BSA) for 1 hour. The cells were stained with primary antibodies overnight at 4°C, followed by incubation of fluorescent dye-conjugated secondary antibodies (Cell Signaling Technology, Danvers, MA). Cells nuclei were stained with DAPI (Sigma Aldrich, MO, USA). Confocal images were obtained with a NIKON confocal microscope.

### Alkaline comet assay

The comet assay was performed using CometAssay Kit according to the manufacturer’s protocol (Trevigen, Gaithersburg, MD). A total of 1×105 cells were resuspended to 1mL with PBS. Mix 10μL cell suspension with 100μL low melting point agarose and spread 50μL onto glass slide. After gelling at 4°C in the dark, the sample was then immersed in Lysis Solution for 1 hour, followed by the immersion in Alkaline Unwinding Solution for 20 minutes at room temperature in the dark. The sample was electrophoresed and washed with water and 70% ethanol. The DNA was stained with SYBR Green and detected using a NIKON confocal microscope. Comet tail moments were quantified using the CASP software.

### Cell viability assay

Cell proliferation assay was performed using the Cell Counting Kit-8 (CCK-8) (Vazyme Biotech, Nanjing, China). Cells were seeded into a 96-well plate. A volume of 10 μL CCK8 solution from the CCK8 kit was added to each well for 2–3 hours of incubation. The plate was measured at 450 nm absorbance with a microplate reader (BioTek Instruments, Winooski, VT).

### Flow cytometry

For cell apoptosis analysis, cells were washed with PBS and stained with Annexin V/PI and analyzed on a CytoFLEX Flow Cytometer (Beckman, Brea, CA).

### Real-time RT-PCR

RNA was extracted by the TRIzol reagent (Invitrogen, Carlsbad, CA) and 500μg of RNA was reverse transcribed to cDNA using Evo M-MLV RT Kit with gDNA Clean for qPCR kit (Accurate Biology, Changsha, China). Real-time RT-PCR was performed with SYBR Green Premix Pro Taq HS qPCR Kit (Accurate Biology, Changsha, China) using the QuantStudio 5 Real Time PCR system (Applied Biosystems). The qRT-PCR primers for TOP2A are listed in S2 Table in S1 File.

### Xenografts

All animal experiments were performed in strict accordance with the ARRIVE guidelines and approved by the Animal Care and Use Committee of Shanghai JiaoTong University School of Medicine. For tumor xenografts experiments, 4×10^6^ NCI-H929 cells were mixed with Corning Matrigel and implanted subcutaneously in the right flank of four-week-old NOG female mice. The mice were randomly assigned to each group. Indisulam was administered intraperitoneally at 10 mg/kg three times a week. Melphalan was administered intraperitoneally at 3 mg/kg once a week diluted in PBS. Tumor diameters were measured using calipers and final tumor volumes were calculated using the formula: “Tumor volume = Length × Width × Width/2”. When the subcutaneous tumor volume of the control group approached 2000 mm3, mice were euthanized. the tumors were removed, washed with PBS and then fixed in 4% PFA for immunohistochemistry.

### Bioinformatics analysis

GSE6477 and GSE46816 datasets were downloaded from the GEO database (http://www.ncbi.nlm.nih.gov/geo/). The gene expression data of MMRF CoMMpass study were downloaded from the University of California Santa Cruz Xena database (https://xena.ucsc.edu). All pan-cancer data were downloaded from the TCGA database (https://portal.gdc.cancer.gov). The R package “clusterProfiler” was used to perform GO enrichment analysis and KEGG pathway enrichment analysis on the differentially expressed genes (DEGs).

### Statistics

R software (Version 4.2.1) was used to perform all bioinformatics analyses, and continuous variables were exhibited as mean ± standard deviation (SD). Wilcoxon rank sum tests were used to compare groups. Chi-square or Fisher’s exact tests were used for statistical analyses between two groups of categorical variables. Data was analyzed and visualized using GraphPad Prism 8. Statistical significance is defined as p < 0.05.

## Results

### Indisulam affects DNA repair pathways and induces DNA damage

**Fig 1:**
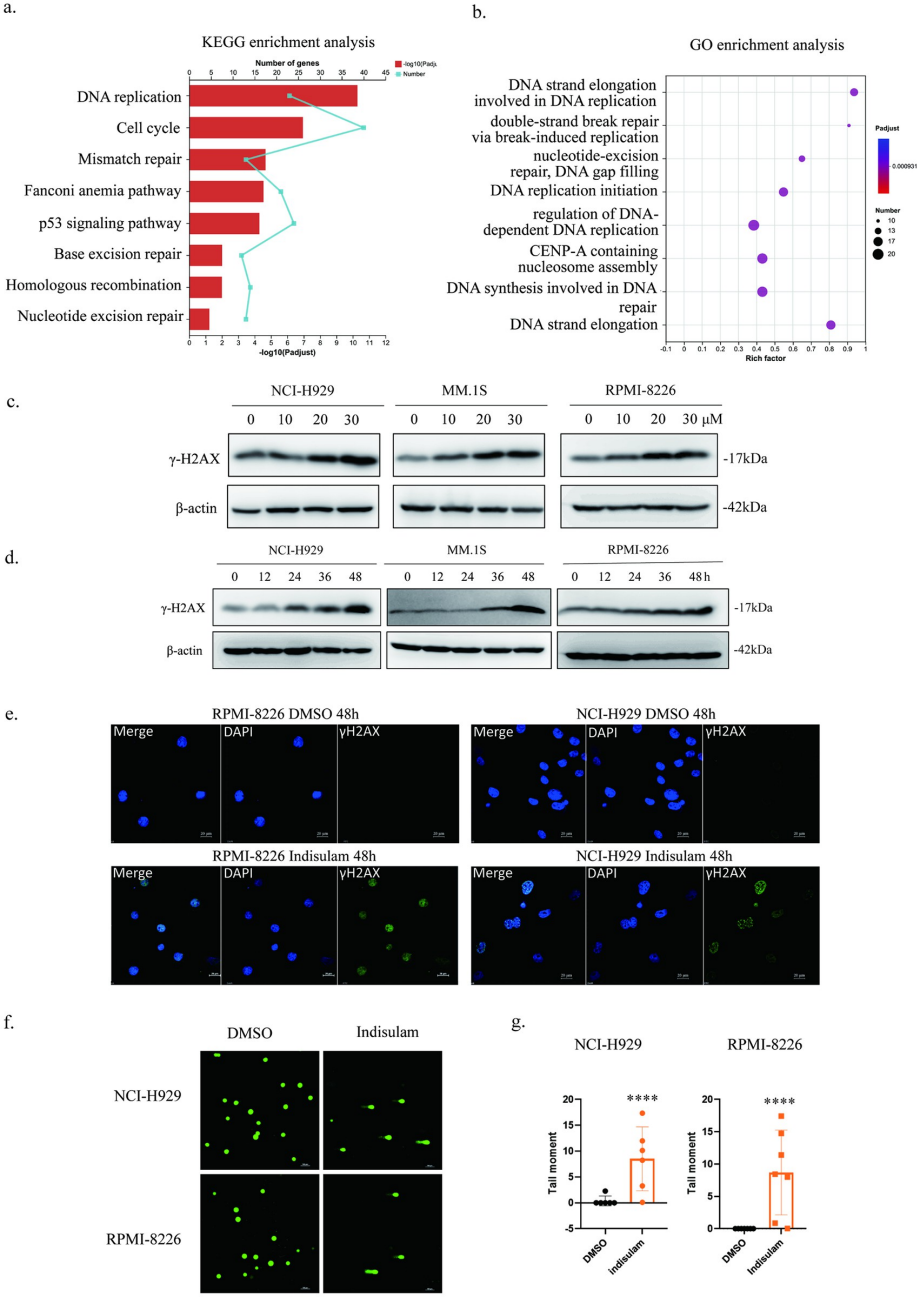
Indisulam affects DNA repair pathways and induced DNA damage a KEGG enrichment analysis of DEGs. b GO enrichment analysis of DEGs c, d NCI-H929, MM.1S and RPMI-8226 cell lines were treated with different concentrations of indisulam for 24h or treated with 20 μM indisulam for various time periods. The final DMSO concentration in each sample is below 0.1%. e NCI-H929 and RPMI-8226 cell lines were treated with 20 μM indisulam for 48h. γH2AX signals were observed in indisulam-treated cells. The experiments were repeated independently three times. f, g NCI-H929 and RPMI-8226 cell lines were treated with 20 μM indisulam for 48h. Comet assay results showed that the DNA fragments were observed in indisulam-treated cells and the tail moments were counted using CASP software. The experiments were repeated independently three times. (*, p < 0.05, **, p < 0.01, ***, p < 0.001, ****, p < 0.0001).

Previous studies of our group have shown that indisulam exerts anti-tumor effects in MM in vitro and in vivo and the IC_50_ for indisulam in MM cell lines ranged from 10 μM to 20 μM. To explore the molecular mechanism of indisulam in MM, we analyzed the RNA-seq data of MM.1S cells treated with DMSO (0.05%) or Indisulam (20 μM) for 48h and investigated the differentially expressed genes (DEGs). KEGG pathway and GO enrichment analysis of the DEGs showed that most of the enriched GO terms and metabolic pathways were involved in DNA repair (Fig 1a and 1b).

To determine whether indisulam caused DNA damage, we treated three MM cell lines with different concentrations of indisulam for 24h and harvested cells at various time points after being treated with 20 μM indisulam. DNA damage-associated protein phosphorylated H2AX (γH2AX) was detected using Western Blotting (WB) and the results confirmed that the levels of γH2AX increased more with the increase of concentration or treatment duration of indisulam (Fig 1c and 1d). As expected, significant DNA damage was observed by γ-H2AX staining and indisulam induced the formation of γ-H2AX foci (Fig 1e). Besides, we measured cellular DNA strand breaks using the alkaline comet assay and found that the tail moment increased dramatically with indisulam treatment (Fig 1f and 1g). Thus, the above results showed that indisulam could induce DNA damage in MM cell lines in a time dependent and concentration dependent manner.

### TOP2A is regulated by indisulam and involved in MM development and progression

**Fig 2:**
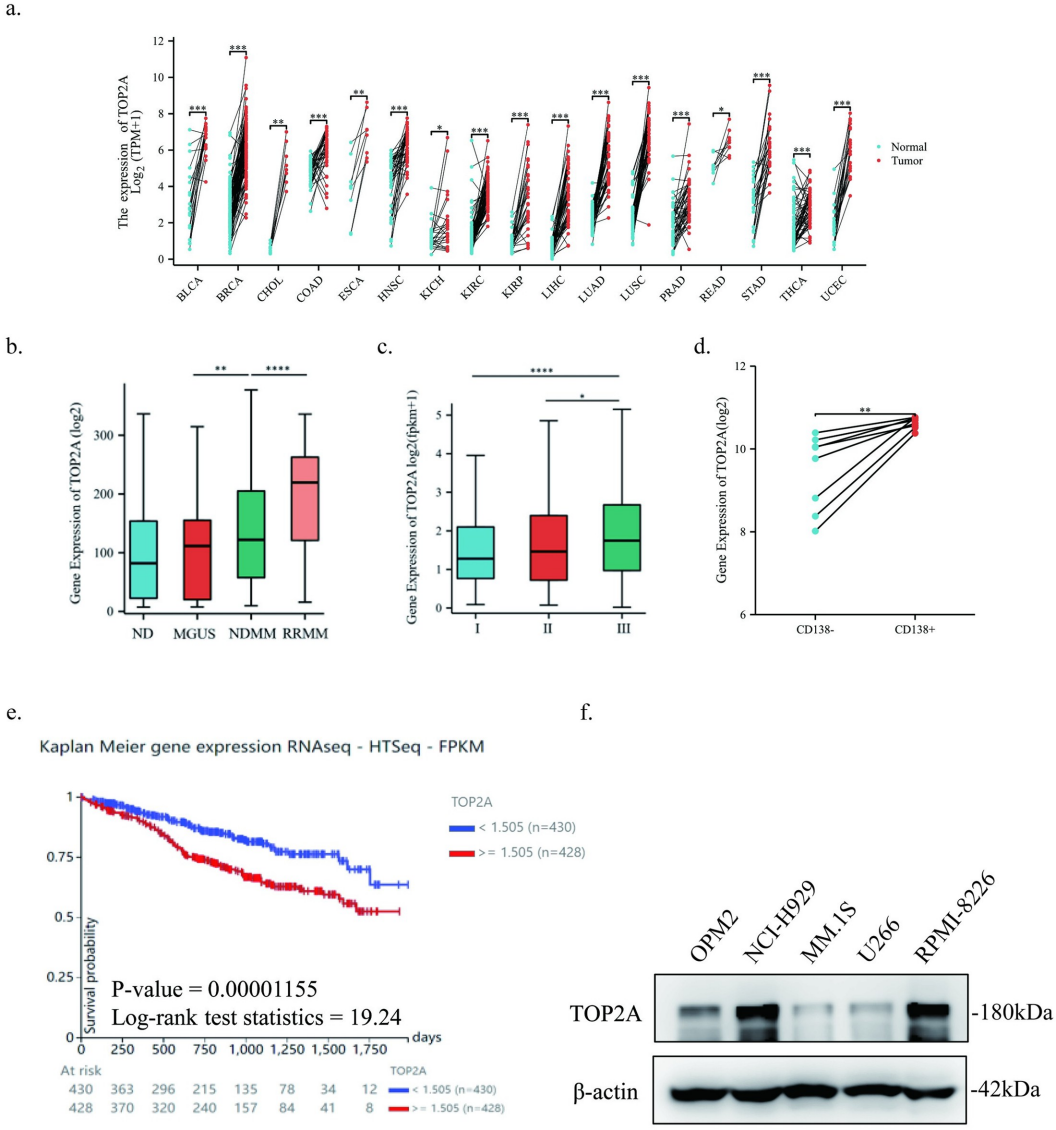
TOP2A is associated with MM development and progression. a TOP2A expression in tumor tissues and their adjacent non-cancerous tissues in the TCGA database. b TOP2A expression in different progression stages in the GSE6477 dataset. c TOP2A expression in different ISS stages in the MMRF-CoMMpass database. d TOP2A expression in CD138− and CD138+ cells in the GSE46816 dataset. e The Kaplan-Meier survival curves in the MMRF-CoMMpass database. f TOP2A expression in five MM cell lines. The experiments were repeated independently three times.

We next sought to investigate the mechanisms by which indisulam induces DNA damage. We selected four genes that were significantly down-regulated and had important roles in DNA damage repair in RNA-seq data. Then, the significance of the four genes in several tumor specimens was assessed using public database. Finally, we selected TOP2A, a gene regulated by indisulam that was involved in DNA repair and had clinical implications. Through the TCGA database, the expression levels of TOP2A were compared between more than twenty tumor tissues and their adjacent non-cancerous tissues, and the results showed that TOP2A was highly expressed in tumor tissues. (Fig 2a, S3 Table in S1 File).

As shown in figures, TOP2A expression was high in MM and increased as the disease progressed (Fig 2b). Besides, a higher expression of TOP2A was significantly associated with a high ISS stage and poor prognosis (Fig 2c and 2e). In a statistical data of eight MM cell lines, TOP2A expression was higher in CD138+ cells than in CD138- cells (Fig 2d). The above results suggested that TOP2A played an important role in MM development and progression.

### Indisulam inhibits the expression of TOP2A by decreasing transcription

**Fig 3:**
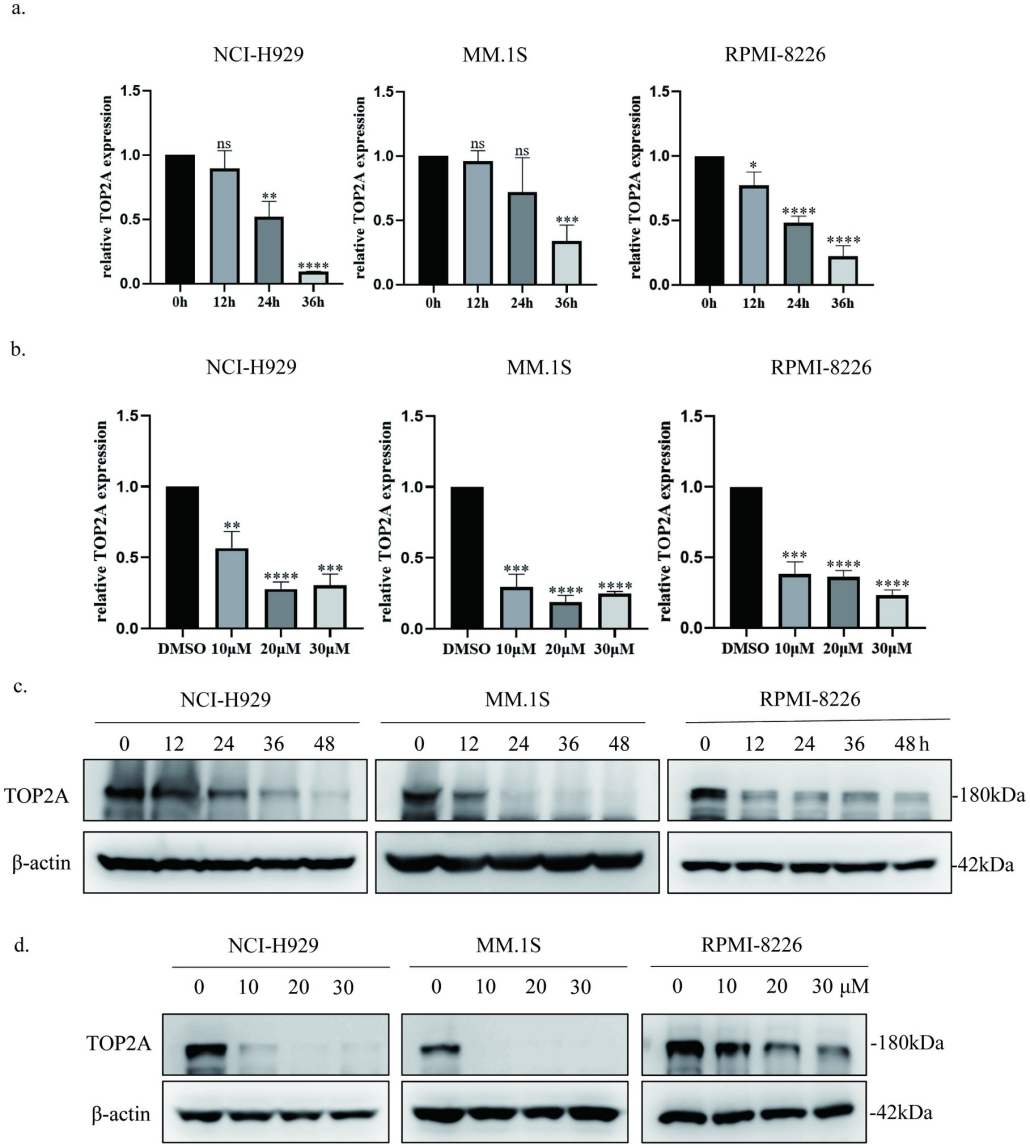
Indisulam inhibits the expression of TOP2A. a, b qPCR analysis of TOP2A mRNA expression. NCI-H929, MM.1S and RPMI-8226 cell lines were treated with different concentrations of indisulam for 24h or treated with 20 μM indisulam for various time periods. The experiments were repeated independently three times. c, d TOP2A protein levels in MM cells. NCI-H929, MM.1S and RPMI-8226 cell lines were treated with different concentrations of indisulam for 24h or treated with 20 μM indisulam for various time periods. The experiments were repeated independently three times. (*, p < 0.05, *, p < 0.01, *, p < 0.001, *, p < 0.0001).

We first examined the protein expression levels of TOP2A in five MM cell lines (NCI-H929, MM.1S, RPMI-8226, U266, and OPM2) and found that NCI-H929 and RPMI-8226 showed higher levels of TOP2A while TOP2A expression in MM.1S and U266 was relatively low (Fig 2f). Therefore, we chose NCI-H929, RPMI-8226 and MM.1S for all subsequent experiments. Next, we detected TOP2A gene expression under different concentration or treatment duration of indisulam. The results confirmed that indisulam decreased the TOP2A mRNA expression as measured by qPCR. TOP2A was then detected using WB and the results indicated that the levels of TOP2A were getting lower with the increase of concentration or treatment duration of indisulam. (Fig 3a–3d). Overall, indisulam could reduce the expression of TOP2A by decreasing transcription.

### Knockdown of TOP2A inhibits MM growth and induces cell apoptosis in association with DNA damage

**Fig 4:**
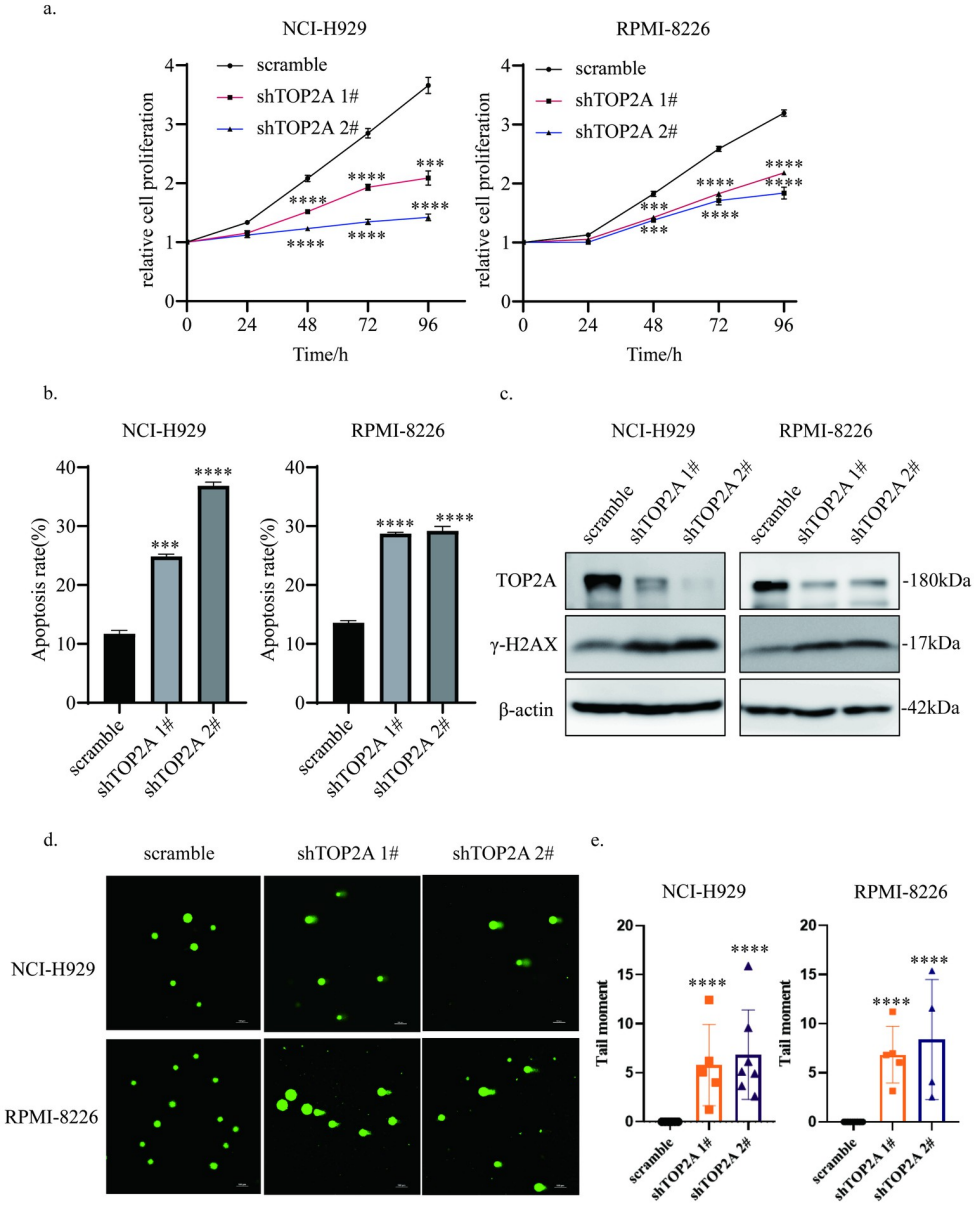
Knockdown of TOP2A inhibits MM growth and induces cell apoptosis in association with DNA damage a Influence of TOP2A knockdown on proliferation in MM cells. The experiment was repeated independently three times. b Influence of TOP2A knockdown on apoptosis in MM cells. The apoptosis rates in NCI-H929 are 11.7±0.57, 24.84±0.42, 36.85±0.62. The apoptosis rates in RPMI-8226 are 13.59±0.36, 28.71±0.22, 29.18±0.76. The experiment was repeated independently three times. c Transfection efficiency of shTOP2A lentiviral particles and γH2AX protein levels. d, e Comet assay results showed that knockdown of TOP2A induced significant DNA breaks and the statistical results were shown in the right panel. The experiment was repeated independently three times. (*, p < 0.05, **, p < 0.01, ***, p < 0.001, ****, p < 0.0001).

To determine the biological function of TOP2A, we designed two lentiviral particles containing shRNA against TOP2A and assessed their efficacy by WB (Fig 4c). The effect of TOP2A knockdown on MM proliferation was measured by CCK8 assay, and the experiment confirmed that TOP2A knockdown inhibited the growth of MM cells. Of note, the efficacy of shTOP2A 2# was stronger than that of shTOP2A 1# in NCI-H929 cells, and the proliferation of NCI-H929 cells was suppressed more significantly when cells were treated with shTOP2A 2# (Fig 4a). In addition, the Annexin V–PI analysis indicated that there was a prominent increase in cell apoptosis on day 4 after shTOP2A lentiviruses transfection (Fig 4b, S1 Fig in S1 File).

Subsequently, we attempted to further investigate whether the declined proliferation was associated with DNA damage. We performed alkaline comet assays, and the tail moment increased dramatically with TOP2A knockdown (Fig 4d and 4e). Besides, the levels of γ-H2AX increased in MM cells treated with shTOP2A lentiviral particles (Fig 4c). Together, the data showed that TOP2A knockdown induced DNA damage leading to reduced MM growth.

### The combination of indisulam and melphalan inhibits MM cell proliferation and induces cell death and DNA damage

The above results indicated that indisulam inhibited the expression of TOP2A which was known as an enzyme essential for DNA repair and participated in relieving DNA topological pressure. We considered that melphalan, a commonly used chemotherapeutic agent for MM, was able to cause DNA damage. We speculated that indisulam was likely to have a synergistic effect with melphalan.

**Fig 5:**
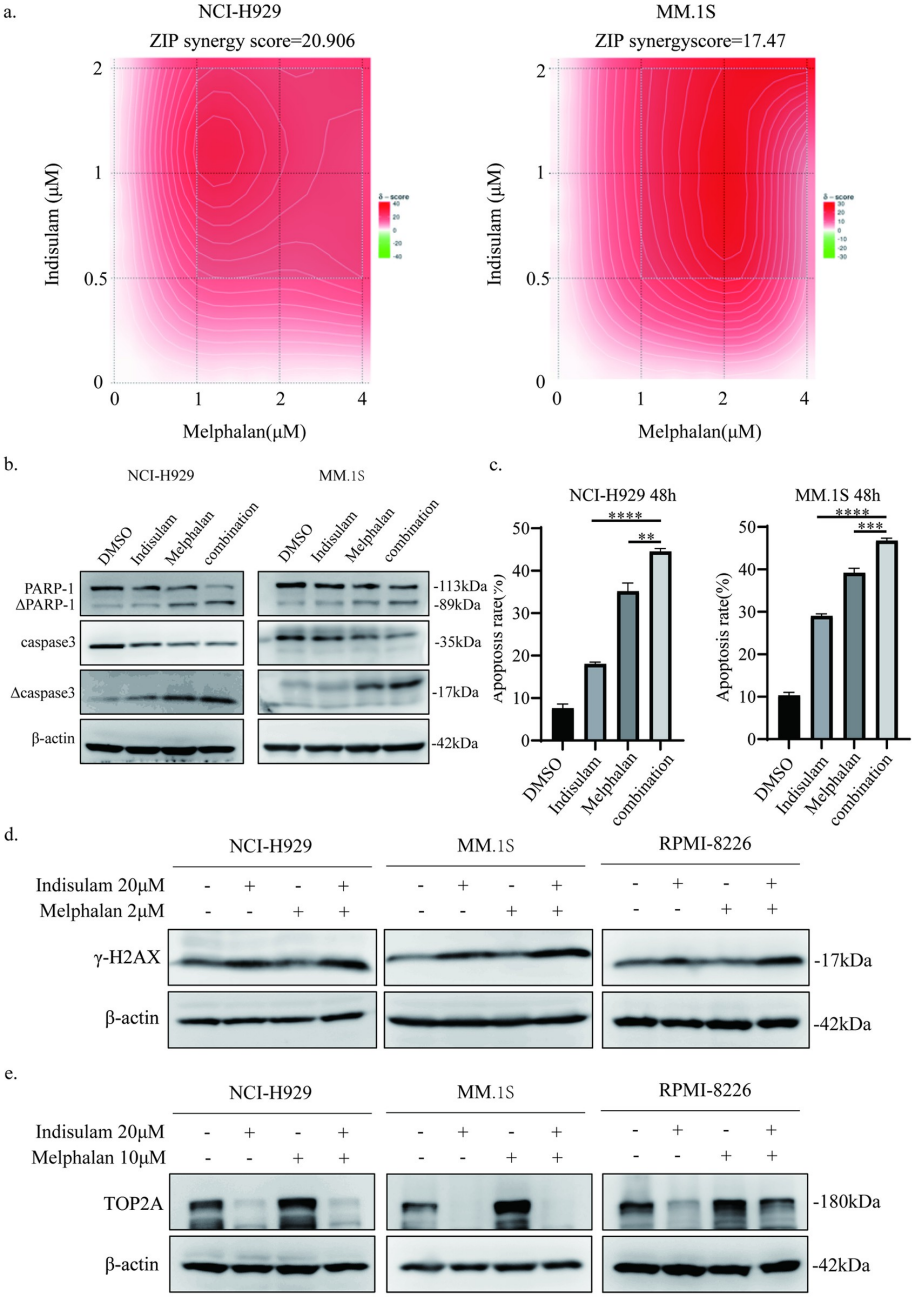
The combination of indisulam and melphalan inhibits MM cell proliferation and induces cell apoptosis and DNA damage. a NCI-H929 and MM.1S cell lines were treated with different concentrations of indisulam or melphalan for 48h. CCK-8 was used to detect cell proliferation and synergy score was calculated by synergyfinder website. The experiment was repeated independently three times. b Apoptosis-related proteins were detected by WB. NCI-H929 and MM.1S cell lines were treated with 20 μM indisulam or 10 μM melphalan for 36h. The experiment was repeated independently three times. c Annexin V/PI analysis showed the apoptosis rates of MM cells treated with 20 μM indisulam and 10 μM melphalan alone and in combination for 48h. The apoptosis rates in NCI-H929 are 7.63±0.99, 18.02±0.44, 35.14±1.9 and 44.51±0.73. The apoptosis rates in MM.1S are 10.32±0.71, 29.02±0.48, 39.19±1.07 and 46.75±0.61. The experiment was repeated independently three times. d TOP2A protein was detected by WB in MM cell lines treated with drugs for 48h. The experiment was repeated independently three times. e γH2AX protein levels of MM cells treated with drugs for 36h. The experiment was repeated independently three times. (*, p < 0.05, **, p < 0.01, ***, p < 0.001, ****, p < 0.0001).

First, we treated MM cells with DMSO or with indisulam or with melphalan or in combination and detected cell viability. (Fig 5a). We calculated the synergy score using the online website synergyfinder (http://synergyfinder.fimm.fi). When the score is higher than 10, it indicates that there is a synergy between the two drugs. The results showed that indisulam had a synergistic effect with melphalan. We then detected apoptosis-related proteins, cleaved-PARP1 and cleaved-caspase3, and found that there was a marked increase in the levels of the two proteins in cells treated with combination therapy (Fig 5b). The Annexin V–PI analysis also showed an increase in the apoptosis rates in the combination group (Fig 5c, S2 Fig in S1 File).

To investigate the mechanism by which the combination treatment inhibits MM proliferation, we thus examined the levels of DNA damage-associated protein. We identified a clear increase in γH2AX levels, suggesting DNA damage increased when MM cells were treated with indisulam and melphalan for 36h (Fig 5d). To further explore the mechanism of an increased DNA damage, we detected the effect of melphalan and indisulam on TOP2A. Melphalan caused a significant increase in TOP2A expression and the effect was reversed by the addition of indisulam (Fig 5e). We considered that cells promoted the expression of TOP2A to repair melphalan-induced DNA damage, and indisulam could aggravated this damage by inhibiting TOP2A expression.

### Combinatorial treatment delays MM growth in vivo

**Fig 6:**
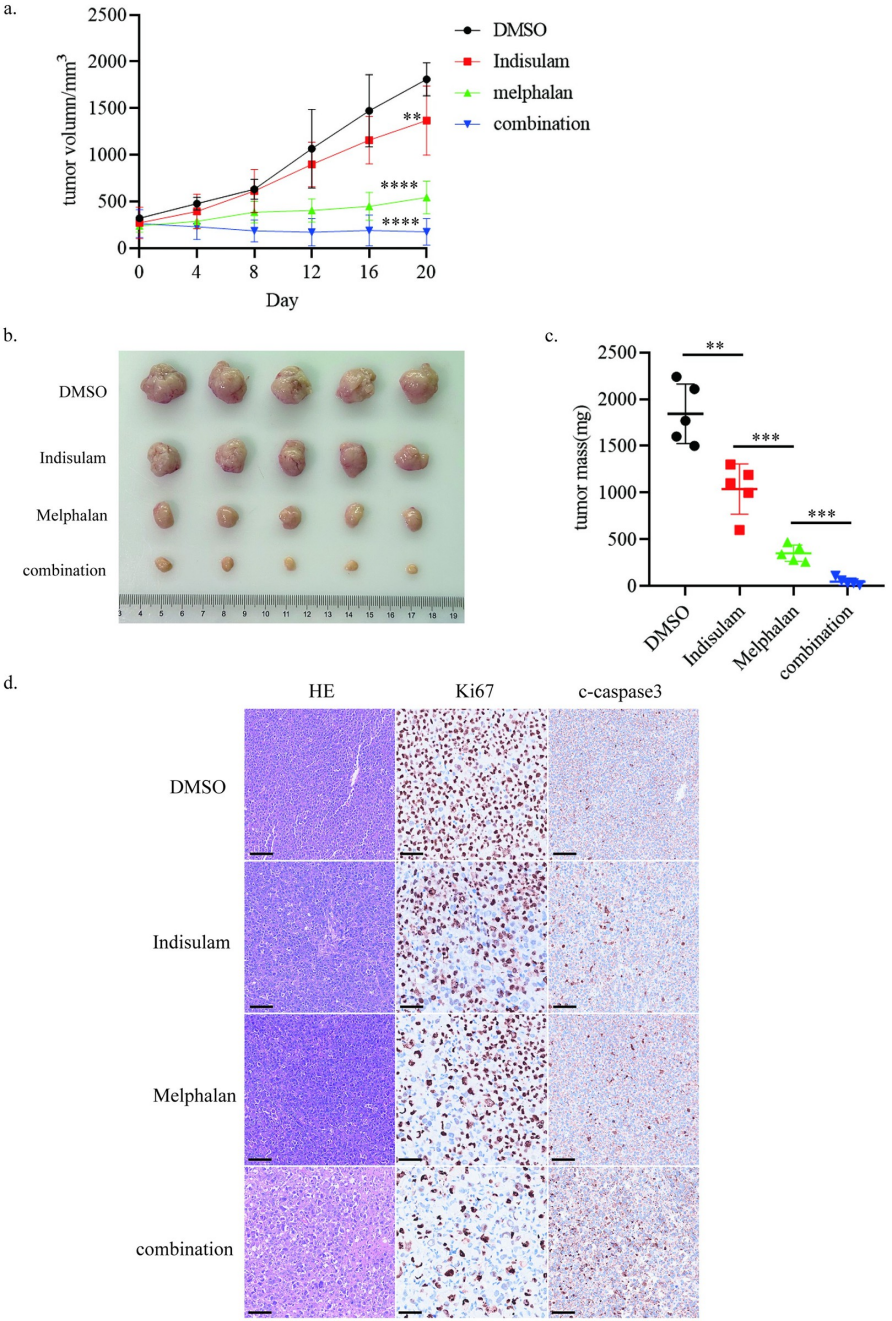
Combinatorial treatment delays MM growth in vivo. a. NCI-H929 cells were implanted subcutaneously in the right flank of NOG mice and the tumor volume was detected every four days. b After 20 days, mice were sacrificed and the tumors were showed. c The tumor weight was measured. d. Immunohistochemistry analysis in tumor tissues. Scale bars: 100μM. (*, p < 0.05, **, p < 0.01, ***, p < 0.001, ****, p < 0.0001).

To further expand on our observations in vitro, we employed an NCI-H929 subcutaneous tumor xenograft model and verified the antitumor efficacy of these drugs alone and in combination. The results showed that single-agent treatment was able to inhibit tumor growth while the combination decreased tumor volume more significantly compared with each agent alone. We then measured the tumor weight and the difference between groups was consistent with the in vitro results. (Fig 6a–6c). Meanwhile, the body weight of mice was monitored, and no noticeable body weight loss of the mice was observed. We also immunestained tumor tissues for Ki-67 and c-caspase3. Ki-67 immunostaining was remarkably reduced and c-caspase3 was apparently increased in the combination group compared with the indisulam group and the melphalan group, confirming that indisulam synergized with melphalan to inhibit tumor growth and promote cell apoptosis (Fig 6d).

## Discussion

Although the use of many new drugs significantly prolongs the overall survival of MM patients, MM remains an incurable disease [21]. Therefore, there is an urgent need for research and development of new drugs and therapeutic strategies to address this issue. Previous studies conducted by our group have demonstrated the significant anti-tumor effects of indisulam in MM. In this study, we investigated the inhibitory effects of indisulam on MM growth by inducing DNA damage through downregulating the expression of TOP2A, and evaluated the preclinical efficacy of combining indisulam with melphalan both in vitro and in vivo.

Topoisomerase IIα (TOP2A) is an enzyme encoded by the TOP2A gene, which is highly expressed in rapidly dividing cells and can promote tumor proliferation and induce metastasis [22, 23]. TOP2A plays a crucial role in DNA replication and repair. It reversibly cleaves DNA duplexes, relieve topological pressure of intracellular DNA, and is essential in sister chromatid separation and the maintenance of chromatin condensation during metaphase [24, 25]. Consistent with previous reports, silencing TOP2A in MM inhibits tumor growth and induces DNA damage.

As a molecular glue, indisulam was reported to link RBM39 to DCAF15 and thus promoted the degradation of RBM39 via the proteasome pathway [10]. In recent years, many studies found that indisulam could induce degradation of various proteins as molecular glue. It can promote the interaction between ZEB1 and DCAF15 and enhance ubiquitination and proteasomal degradation of ZEB1 [12], and induce the degradation of PRPF39 via DCAF15 [26]. Our results showed that indisulam could also reduce the expression of TOP2A by promoting degradation via the proteasome pathway (S3 Fig in S1 File). RBM39 is highly expressed in most cancers and inhibition of its function causes lethal damage in a variety of cancers including lung, breast, and colorectal cancers [27–29]. Inhibiting RBM39 can cause changes in numerous downstream proteins. To explore whether decreased RBM39 affects TOP2A and DNA damage, we detected TOP2A and γ-H2AX after knockdown of RBM39 in MM cells, and found that there were no changes on both proteins. Indisulam may regulate TOP2A through pathways which is independent of RBM39. We speculate that indisulam may also be involved in TOP2A regulation as a molecular glue and we will conduct further experimental validation in the future.

Melphalan is commonly used in the treatment of MM and acts as an alkylating agent to bind N7 of the guanine base of DNA and cause DNA double-strand cross-link. It inhibits DNA replication and increases the topological pressure of DNA in MM cells, causing cell cycle arrest and cell death. The activation of DNA repair pathways is an essential mechanism of resistance to melphalan. A study showed that the interference with TOP2A potentiated melphalan cytotoxicity in lung fibroblasts [30], which was consistent with our research. We conjectured that indisulam could have a synergistic effect with melphalan by reducing the expression of TOP2A and weakening the compensation of MM cells to melphalan-induced DNA damage.

In this study, we discovered that indisulam regulated TOP2A at both the transcriptional level and through the proteasome degradation pathway, leading to DNA damage in MM cells. Additionally, we propose a novel finding that indisulam exhibits a synergistic effect with melphalan. Our findings provide a new idea for the clinical treatment of MM.

## Supporting information

S1 File (DOCX)

S1 Raw images (PDF)
