# Supplementary material for: Indisulam synergizes with melphalan to inhibit Multiple Myeloma malignancy via targeting TOP2A
Source: PLoS One. 2024 Apr 9;19(4):e0299019. doi: 10.1371/journal.pone.0299019 (PMC11003618; doi:10.1371/journal.pone.0299019)
Supplement: S1 File — (DOCX) [file pone.0299019.s001.docx]

**Indisulam Synergizes with Melphalan to Inhibit Multiple Myeloma Malignancy via Targeting TOP2A**

**Table S1. Primary antibodies used for western blotting analysis or Immuno-histochemistry**

| **Antibodies** | **Manufacturer** | **Catalog#** | **Dilution** |
| --- | --- | --- | --- |
| TOP2A | Abclonal | 1 | 1:1000 |
| Caspase-3 | Cell Signaling Technology | #9662 | 1:1000 |
| PARP  γ-H2AX  Ki-67  c-Caspase-3 | Cell Signaling Technology  Abclonal  Cell Signaling Technology  Cell Signaling Technology | #9532  A16440  #9129  #9664 | 1:1000  1:1000  1:400  1:400 |

**Table S2.** **shTOP2A sequences and the sequences of RT-PCR primers**

| NO. | sequences |
| --- | --- |
| shTOP2A 1#: F: | 5’-CCGGGCCTGATTTGTCTAAGTTTAACTCGAGTTAAACTTAGACAAATCAGGCTTTTTG-3’ |
| shTOP2A 1#: R: | 5’-AATTCAAAAAGCCTGATTTGTCTAAGTTTAACTCGAGTTAAACTTAGACAAATCAGGC-3’ |
| shTOP2A 2#: F: | 5’-CCGGGCTCCAAATCAATATGTGATTCTCGAGAATCACATATTGATTTGGAGCTTTTTG-3’ |
| shTOP2A 2#: R: | 5’-AATTCAAAAAGCTCCAAATCAATATGTGATTCTCGAGAATCACATATTGATTTGGAGC-3’ |
| QPCR-TOP2A: F: | 5’-ACCATTGCAGCCTGTAAATGA-3’ |
| QPCR-TOP2A: R: | 5’-GGGCGGAGCAAAATATGTTCC-3’ |

**Table S3.** **Abbreviations and their full names**

| **Abbreviations** | **Full name** |
| --- | --- |
| BLCA | Bladder Urothelial Carcinoma |
| BRCA  CHOL  COAD  ESCA  HNSC  KICH  KIRC  KIRP  LIHC  LUAD  LUSC  PRAD  READ  STAD  THCA | Breast invasive carcinoma  Cholangio carcinoma  Colon adenocarcinoma  Esophageal carcinoma  Head and Neck squamous cell carcinoma  Kidney Chromophobe  Kidney renal clear cell carcinoma  Kidney renal papillary cell carcinoma  Liver hepatocellular carcinoma  Lung adenocarcinoma  Lung squamous cell carcinoma  Prostate adenocarcinoma  Rectum adenocarcinoma  Stomach adenocarcinoma  Thyroid carcinoma |
| UCEC | Uterine Corpus Endometrial Carcinoma |

**Figure S1.**


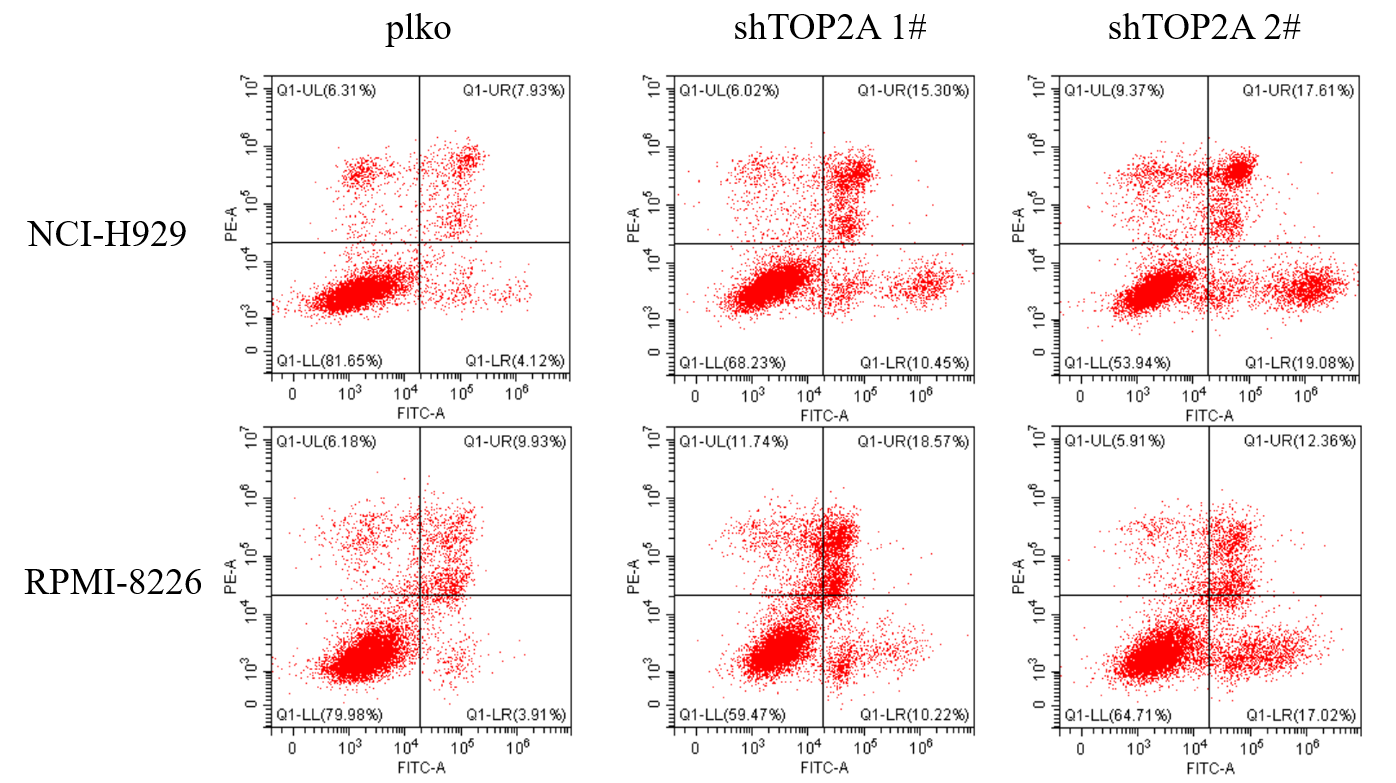


Fig S1 Knockdown of TOP2A induces cell apoptosis in MM cells. NCI-H929 and RPMI-8226 cell lines were harvested 48h after TOP2A knockdown. Apoptosis rates in the indicated groups were assessed via Annexin V/PI assay. The apoptosis rates in NCI-H929 are 11.7±0.57, 24.84±0.42, 36.85±0.62. The apoptosis rates in RPMI-8226 are 13.59±0.36, 28.71±0.22, 29.18±0.76. The experiments were repeated independently three times.

**Figure S2.**


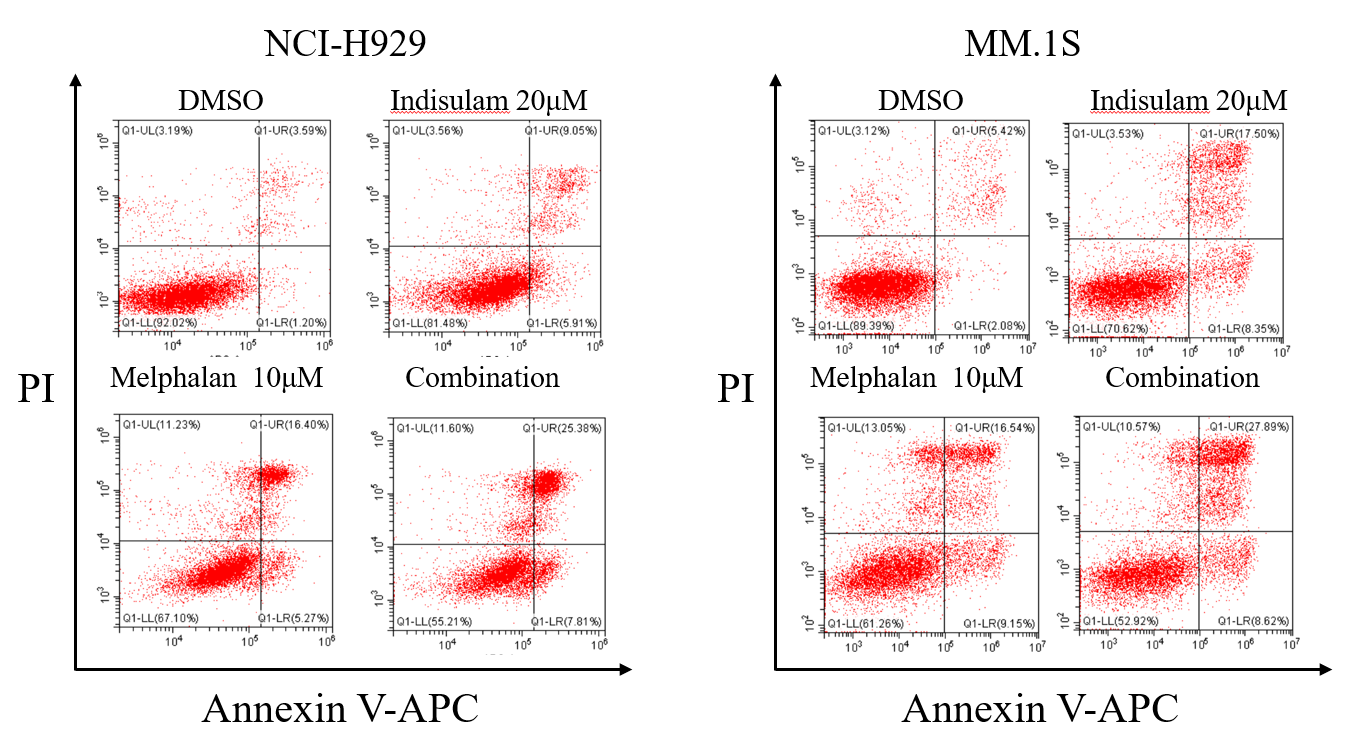


Fig S2 The combination of Indisulam and Melphalan induces cell apoptosis. MM cells were treated with 20 μM indisulam and 10 μM melphalan alone and in combination for 48h. Apoptosis rates were assessed via Annexin V/PI assay. The apoptosis rates in NCI-H929 are 7.63±0.99, 18.02±0.44, 35.14±1.9 and 44.51±0.73. The apoptosis rates in MM.1S are 10.32±0.71, 29.02±0.48, 39.19±1.07 and 46.75±0.61. The experiment was repeated independently three times.

**Figure S3.**


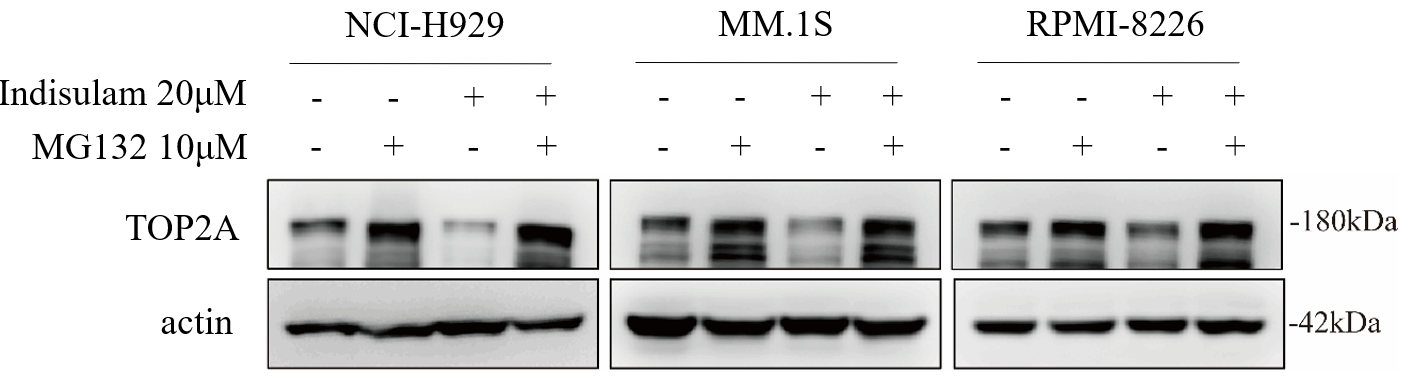


Fig S3 TOP2A might be degraded through the proteasome pathway. NCI-H929, MM.1S and RPMI-8226 cell lines were treated with 20 μM indisulam or 10 μM MG132 for 12h. The final DMSO concentration in each sample is below 0.1%. The experiments were repeated independently three times.
