# Supplementary material for: Indisulam synergizes with melphalan to inhibit Multiple Myeloma malignancy via targeting TOP2A
Source: PLoS One. 2024 Apr 9;19(4):e0299019. doi: 10.1371/journal.pone.0299019 (PMC11003618; doi:10.1371/journal.pone.0299019)
Supplement: S1 Raw images — (PDF) [file pone.0299019.s002.pdf]

Chemiluminescence was detected using ECL reagent and the images were acquired using a chemiluminescence imager. Gel images were acquired using a confocal microscope.

Figure 1c

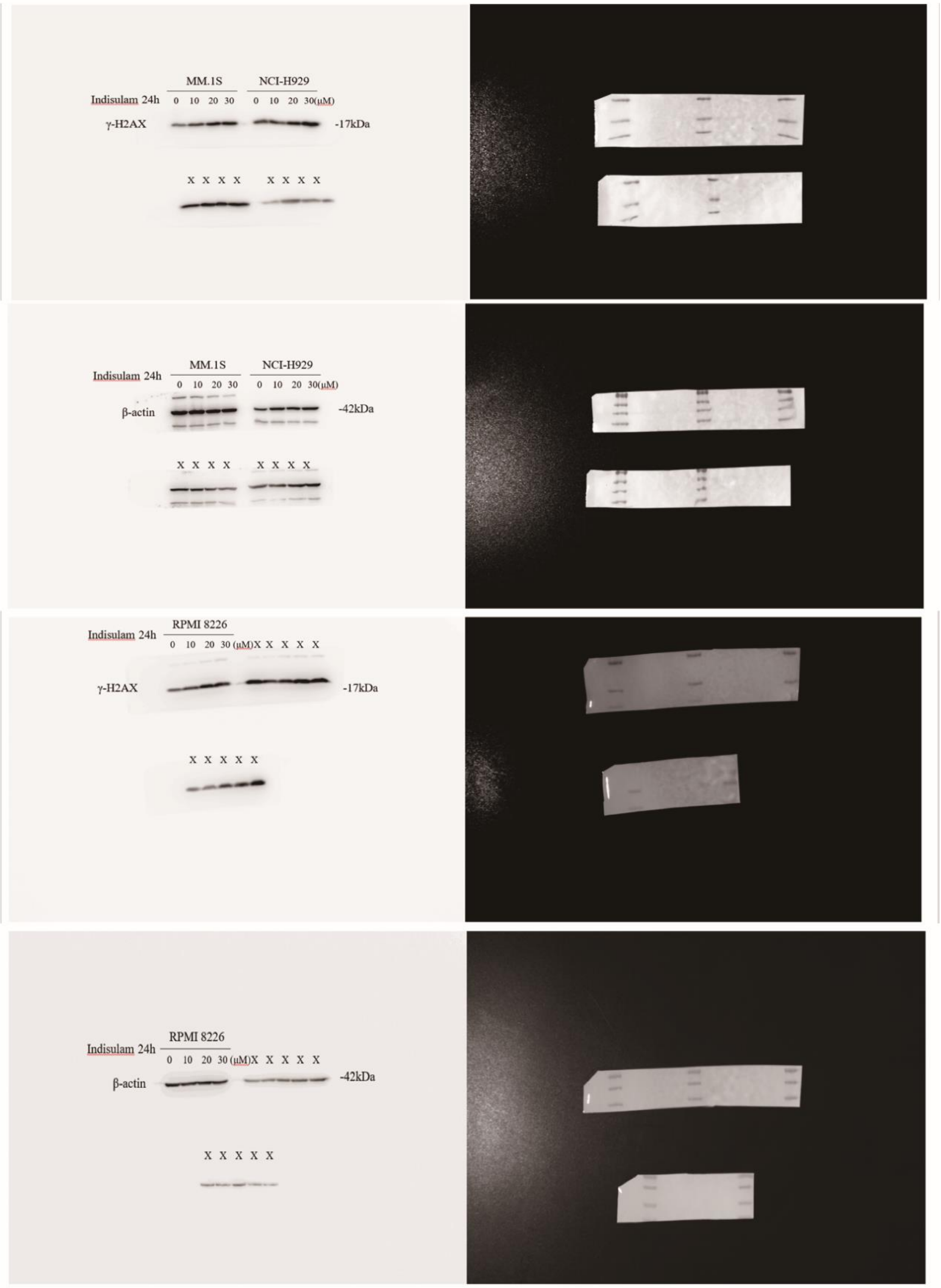

Figure 1d

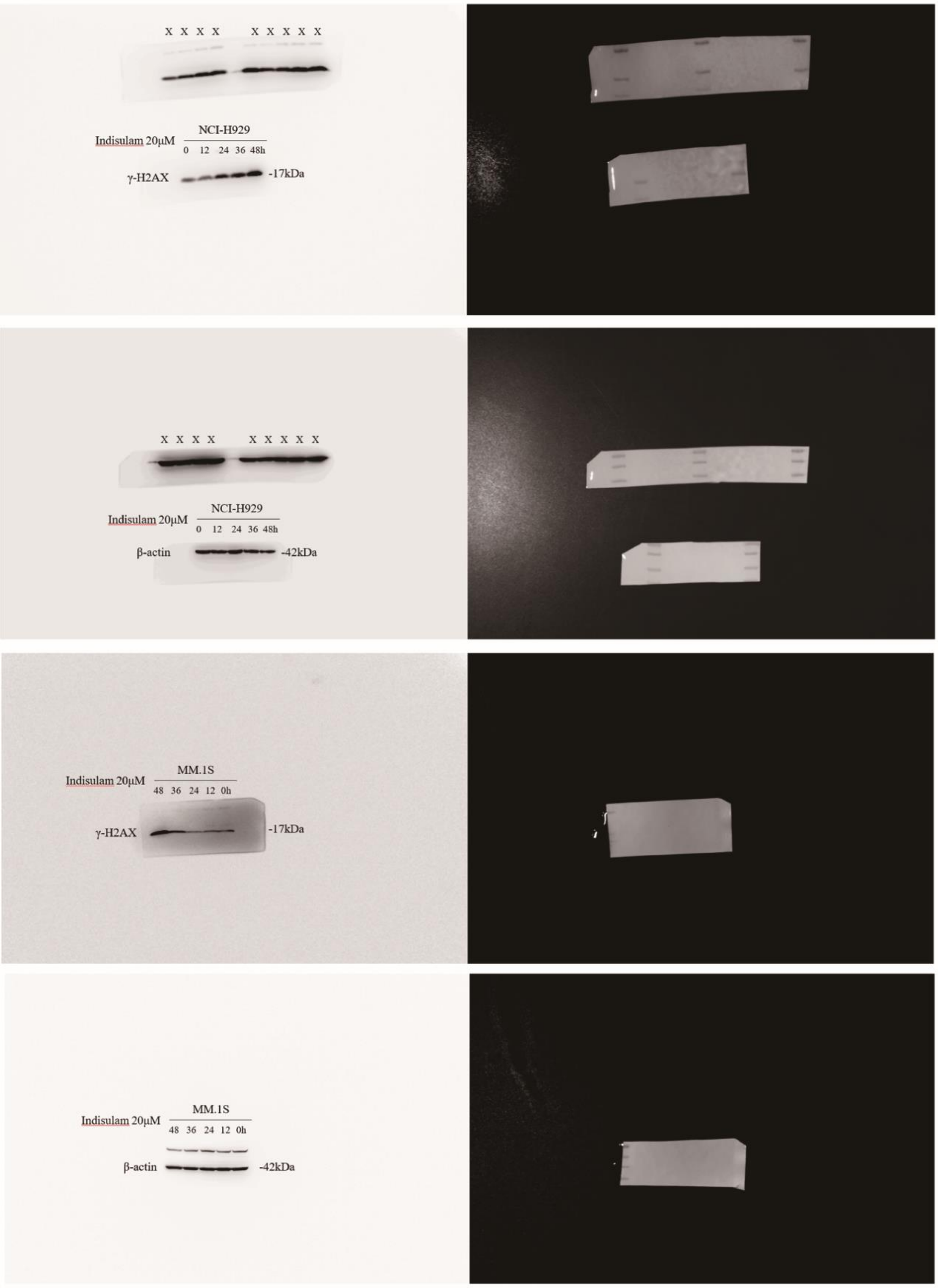
X X X X X X X X X
Indisulam 20μM
NCI-H929
0 12 24 36 48h
γ-H2AX
-17kDa
X X X X X X X X X
Indisulam 20μM
NCI-H929
0 12 24 36 48h
β-actin
-42kDa
Indisulam 20μM
MM.1S
48 36 24 12 0h
γ-H2AX
-17kDa
Indisulam 20μM
MM.1S
48 36 24 12 0h
β-actin
-42kDa

Figure 1d

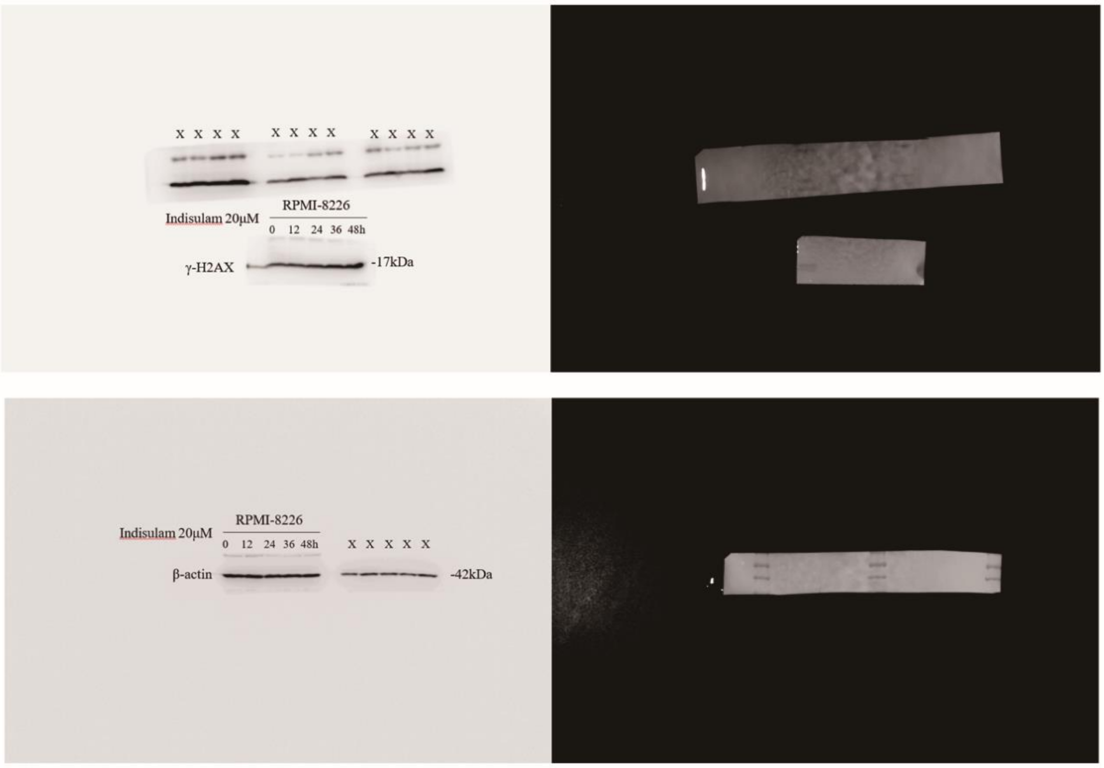

Figure 2f

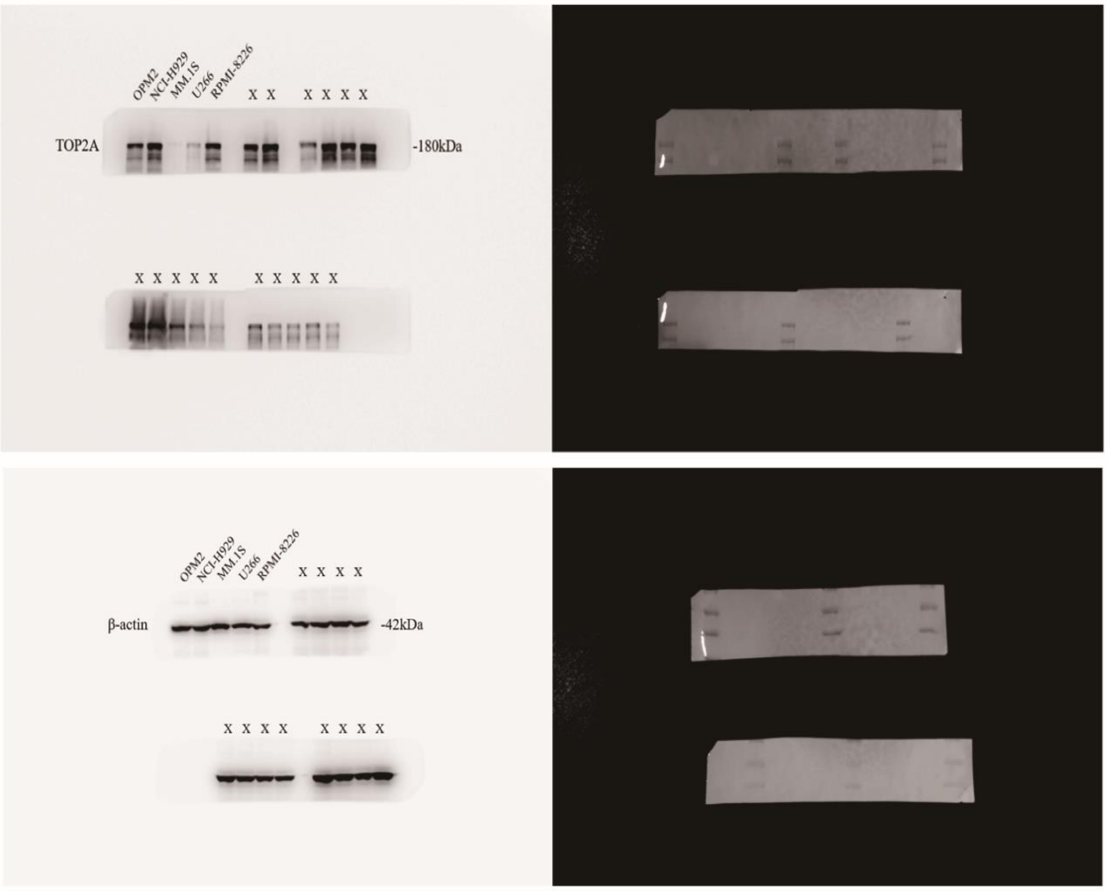

Figure 3c

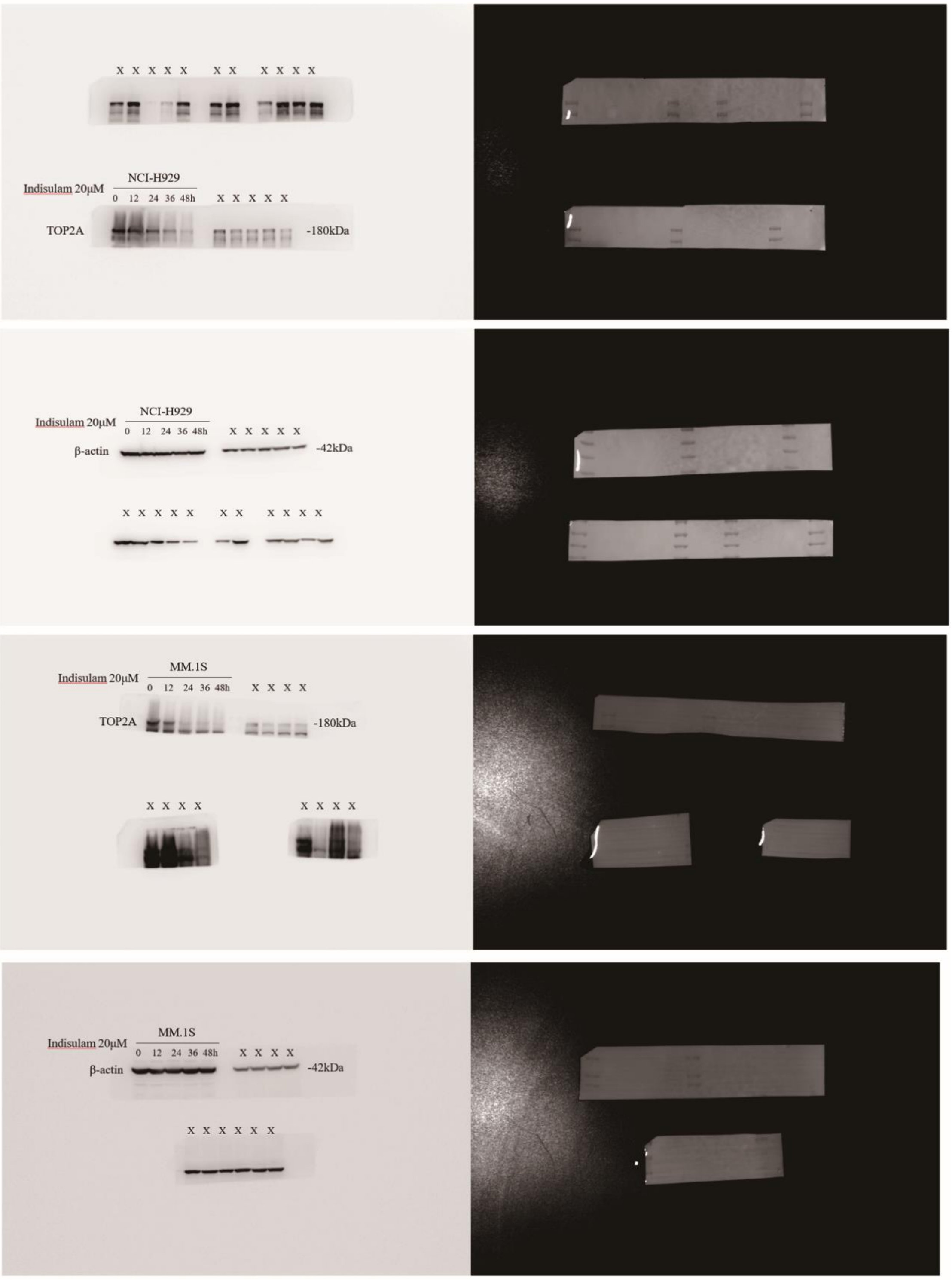

Indisulam 20μM
NCI-H929
0 12 24 36 48h
TOP2A
-180kDa
Indisulam 20μM
NCI-H929
0 12 24 36 48h
β-actin
-42kDa
Indisulam 20μM
MM.1S
0 12 24 36 48h
TOP2A
-180kDa
Indisulam 20μM
MM.1S
0 12 24 36 48h
β-actin
-42kDa

Figure 3c

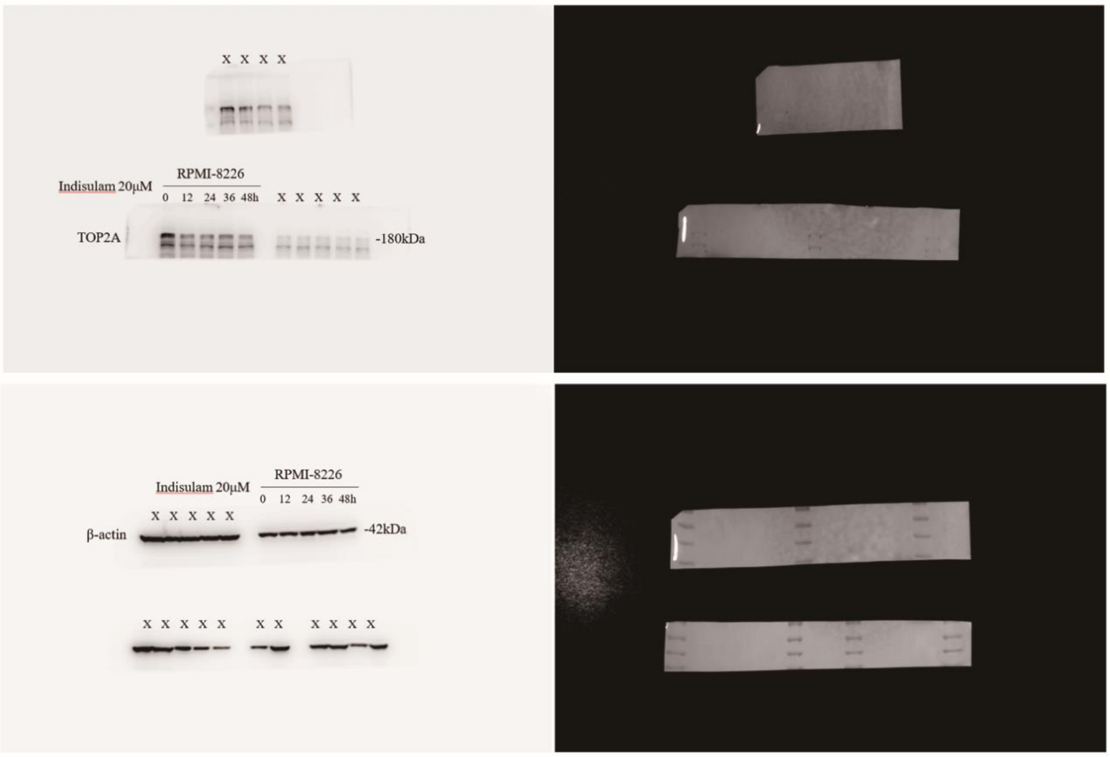
X X X X
Indisulam 20μM
RPMI-8226
0 12 24 36 48h
X X X X X
TOP2A
-180kDa
Indisulam 20μM
RPMI-8226
0 12 24 36 48h
X X X X X
β-actin
-42kDa
X X X X X X X X X X X

Figure 3d

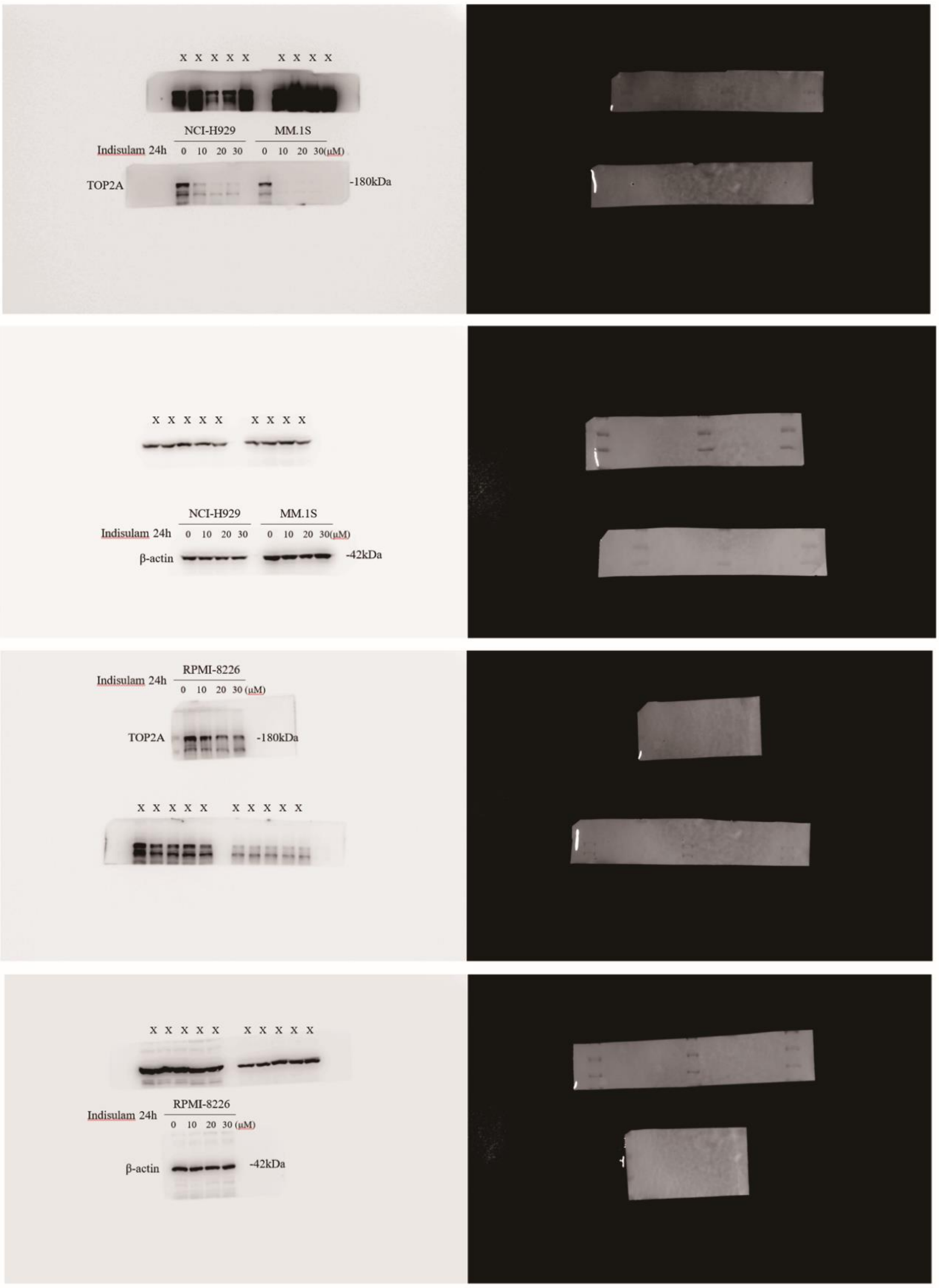
x x x x x x x x x
NCI-H929
MM.1S
Indisulam 24h
0 10 20 30 0 10 20 30(μM)
TOP2A
-180kDa
x x x x x x x x x
NCI-H929
MM.1S
Indisulam 24h
0 10 20 30 0 10 20 30(μM)
β-actin
-42kDa
Indisulam 24h
RPMI-8226
0 10 20 30 (μM)
TOP2A
-180kDa
x x x x x x x x x x
x x x x x x x x x x
Indisulam 24h
RPMI-8226
0 10 20 30 (μM)
β-actin
-42kDa

Figure 4c

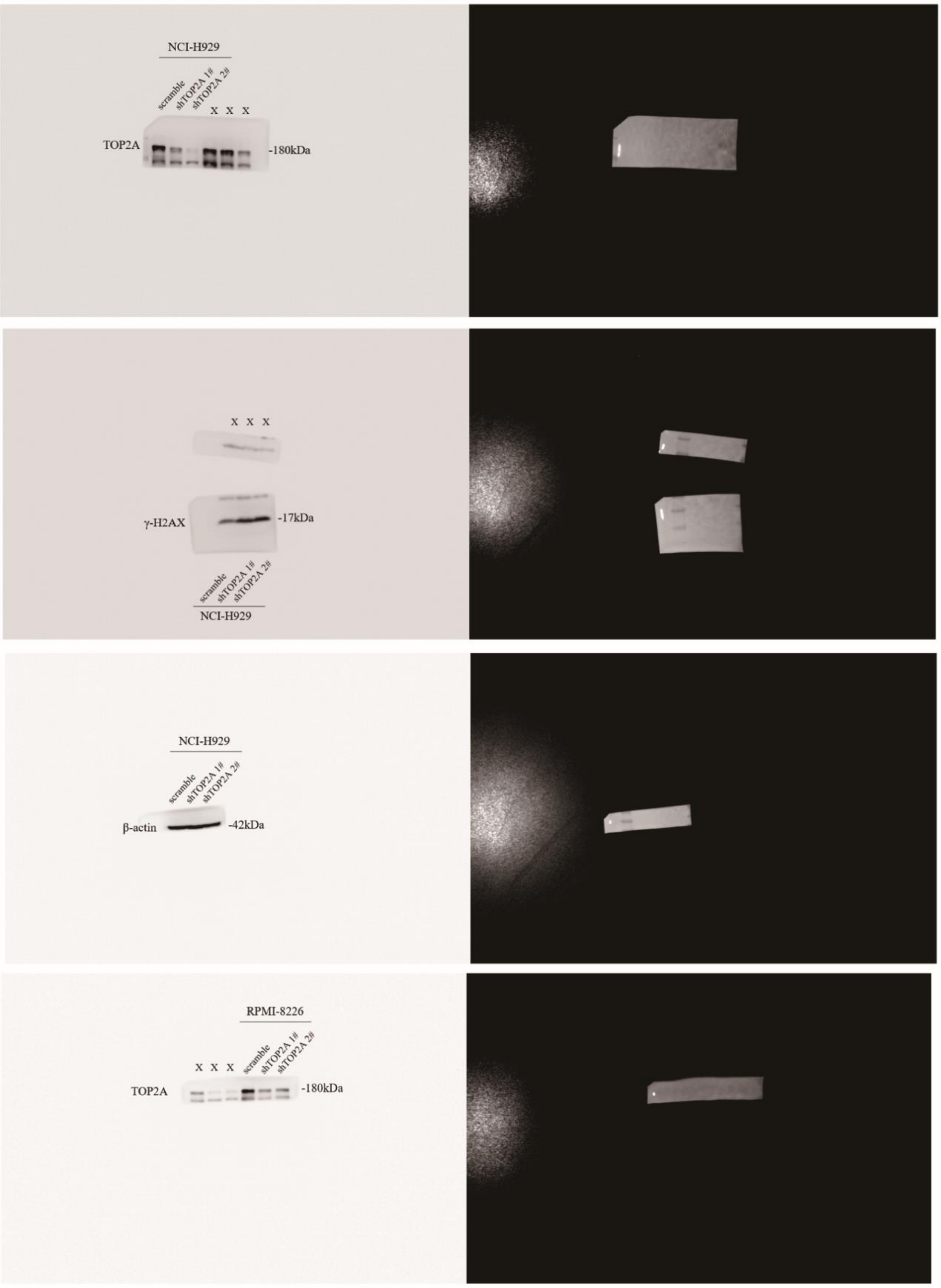

NCI-H929
scramble
shTOP2A 1#
shTOP2A 2#
X X X
TOP2A
-180kDa
X X X
γ-H2AX
-17kDa
scramble
shTOP2A 1#
shTOP2A 2#
NCI-H929
NCI-H929
scramble
shTOP2A 1#
shTOP2A 2#
β-actin
-42kDa
RPMI-8226
X X X
scramble
shTOP2A 1#
shTOP2A 2#
TOP2A
-180kDa

Figure 4c

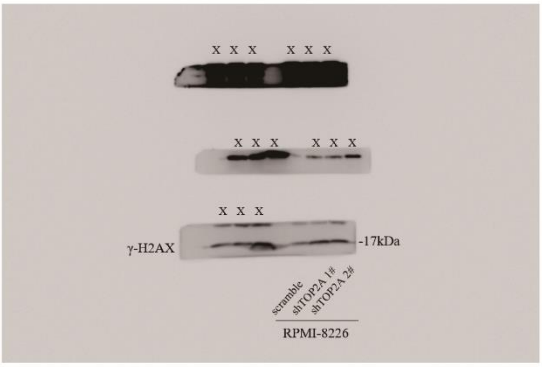

X X X X X X
X X X X X X
X X X
γ-H2AX
-17kDa
scramble
shTOP2A 1#
shTOP2A 2#
RPMI-8226

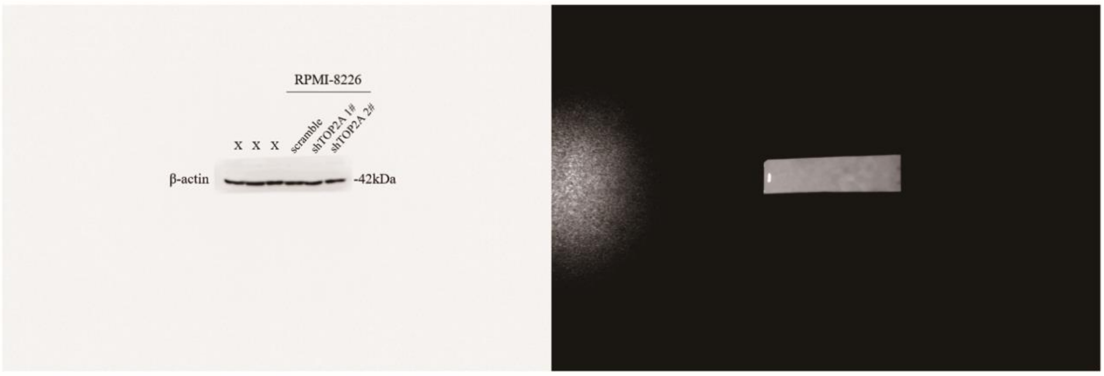

RPMI-8226
X X X
scramble
shTOP2A 1#
shTOP2A 2#
β-actin
-42kDa

Figure 5b

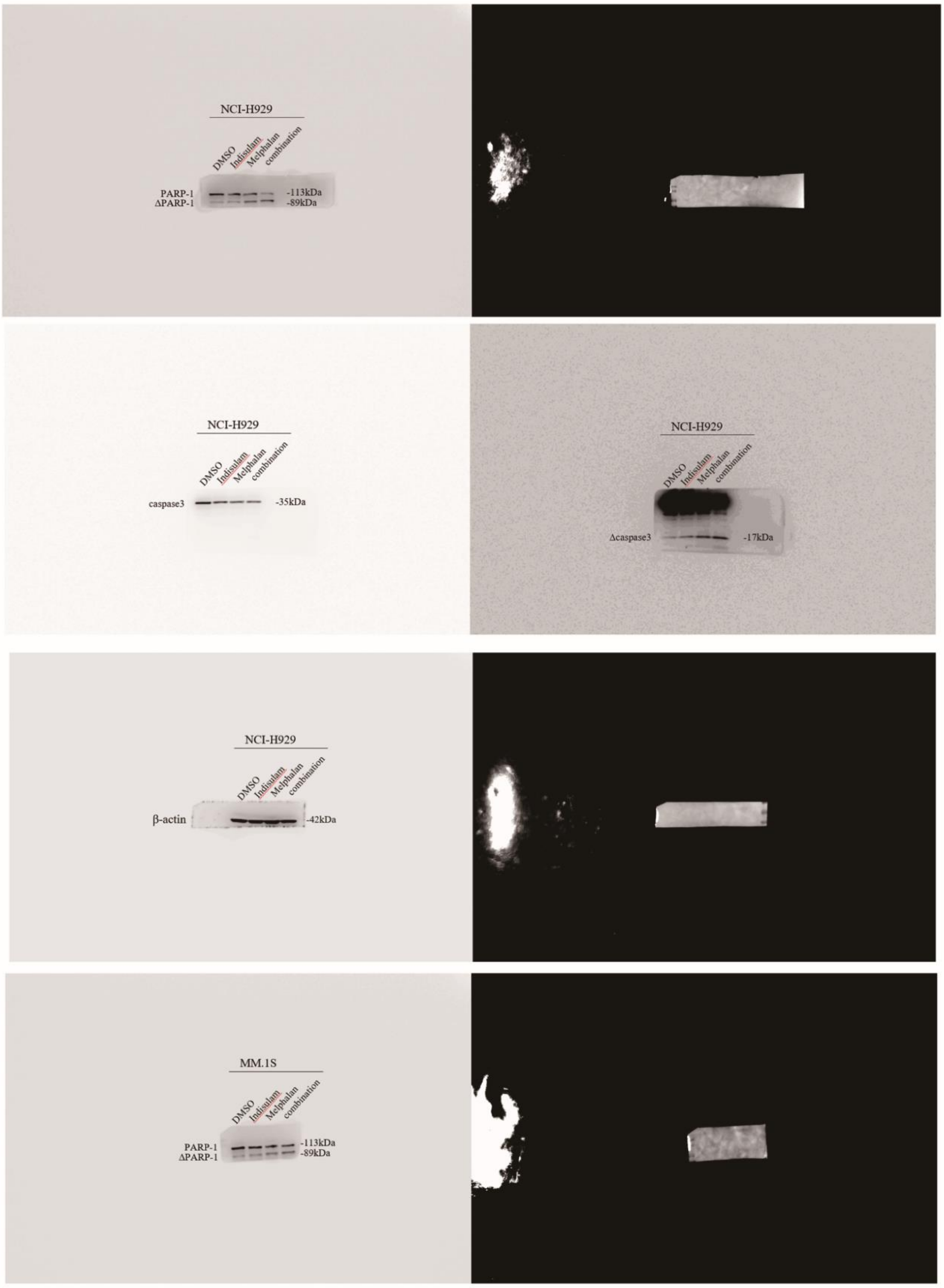
NCI-H929
DMSO
Indisulam
Melphalan
combination
PARP-1
ΔPARP-1
-113kDa
-89kDa
NCI-H929
DMSO
Indisulam
Melphalan
combination
caspase3
-35kDa
NCI-H929
DMSO
Indisulam
Melphalan
combination
Δcaspase3
-17kDa
NCI-H929
DMSO
Indisulam
Melphalan
combination
β-actin
-42kDa
MM.1S
DMSO
Indisulam
Melphalan
combination
PARP-1
ΔPARP-1
-113kDa
-89kDa

Figure 5b

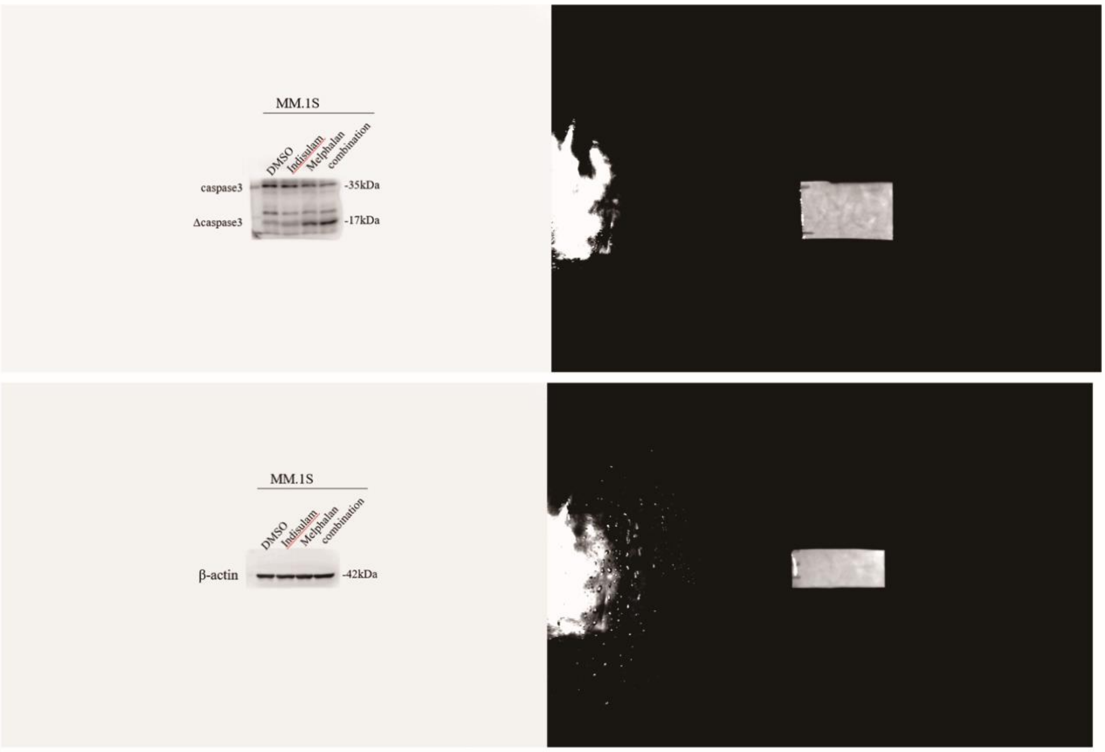
MM.1S
DMSO
Indisulam
Melphalan
combination
caspase3
Δcaspase3
-35kDa
-17kDa
MM.1S
DMSO
Indisulam
Melphalan
combination
β-actin
-42kDa

Figure 5d

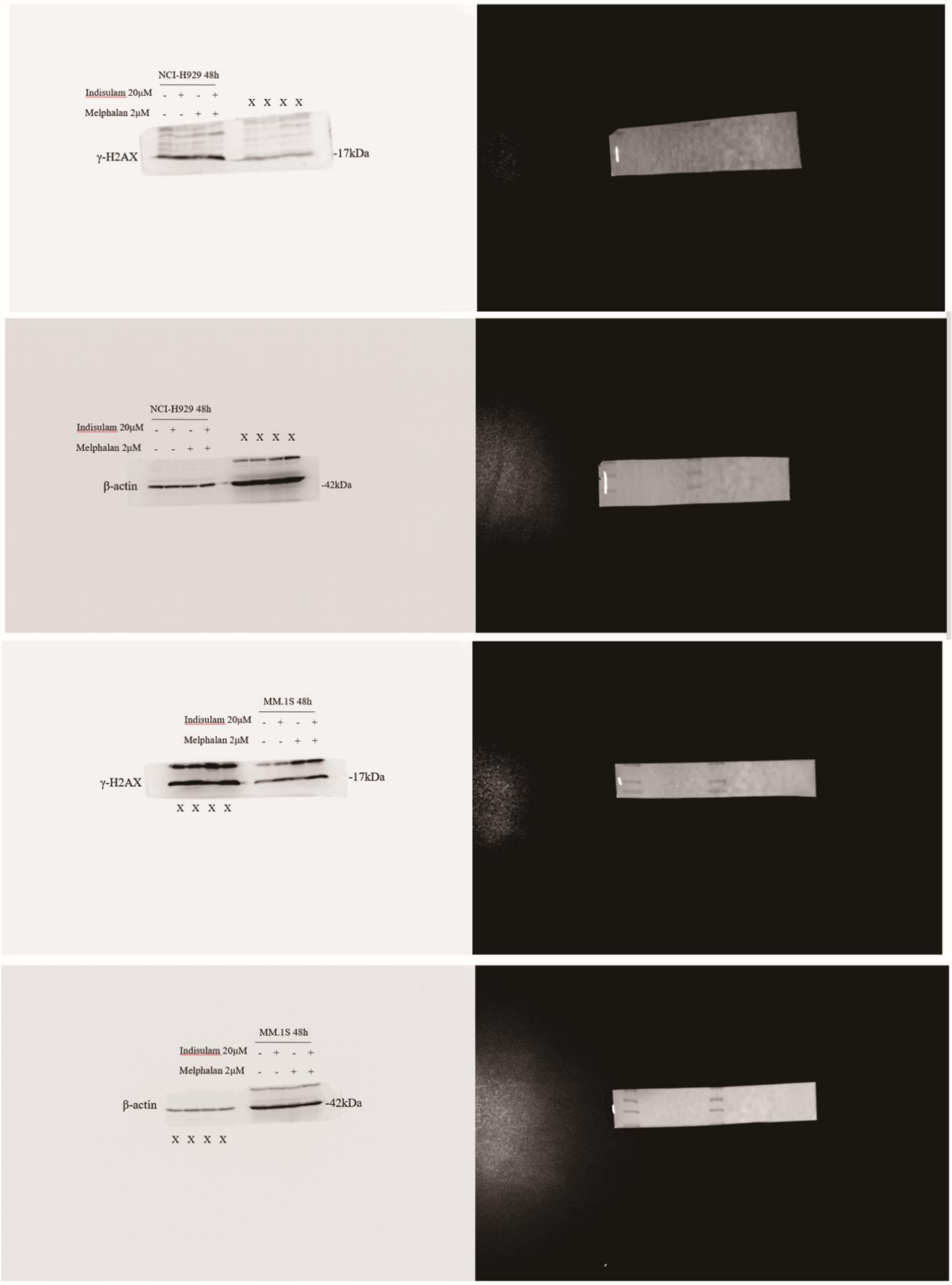
NCI-H929 48h
Indisulam 20μM - + - +
Melphalan 2μM - - + +
X X X X
γ-H2AX
-17kDa
NCI-H929 48h
Indisulam 20μM - + - +
Melphalan 2μM - - + +
X X X X
β-actin
-42kDa
MM.1S 48h
Indisulam 20μM - + - +
Melphalan 2μM - - + +
γ-H2AX
-17kDa
X X X X
MM.1S 48h
Indisulam 20μM - + - +
Melphalan 2μM - - + +
β-actin
-42kDa
X X X X

Figure 5d

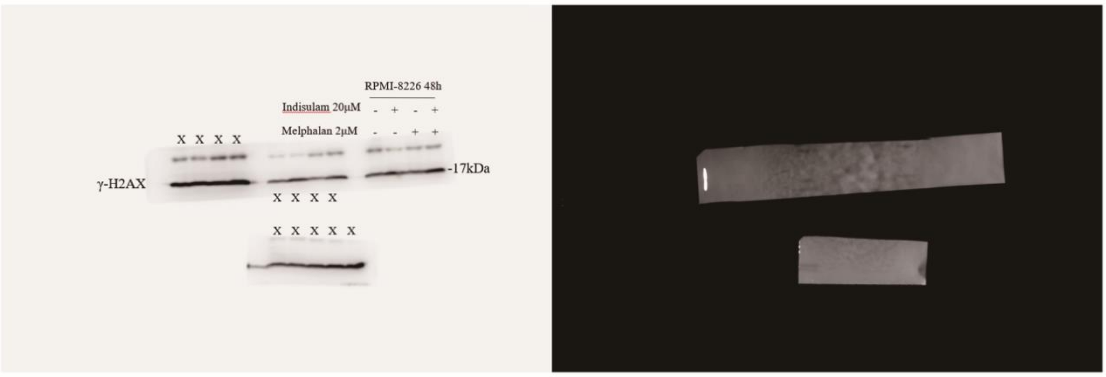

RPMI-8226 48h
Indisulam 20μM - + - +
Melphalan 2μM - - + +
γ-H2AX
-17kDa

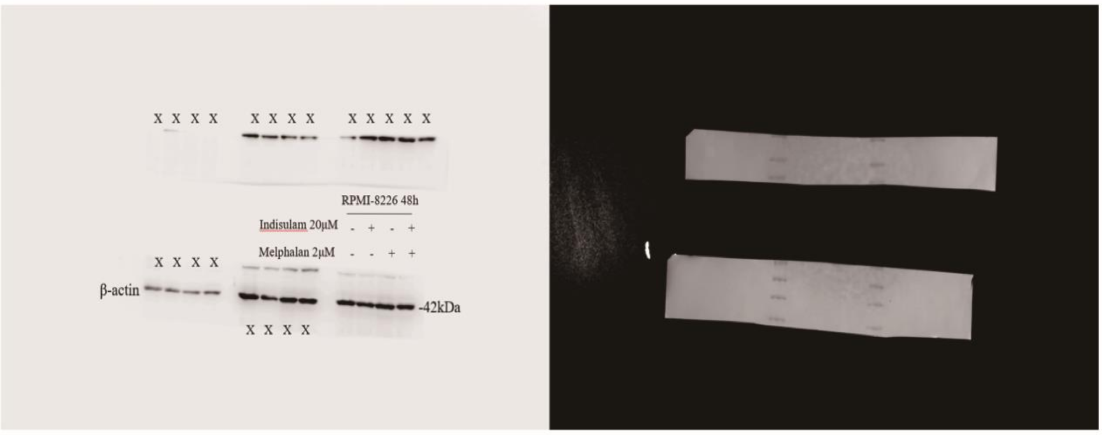

RPMI-8226 48h
Indisulam 20μM - + - +
Melphalan 2μM - - + +
β-actin
-42kDa

Figure 5e

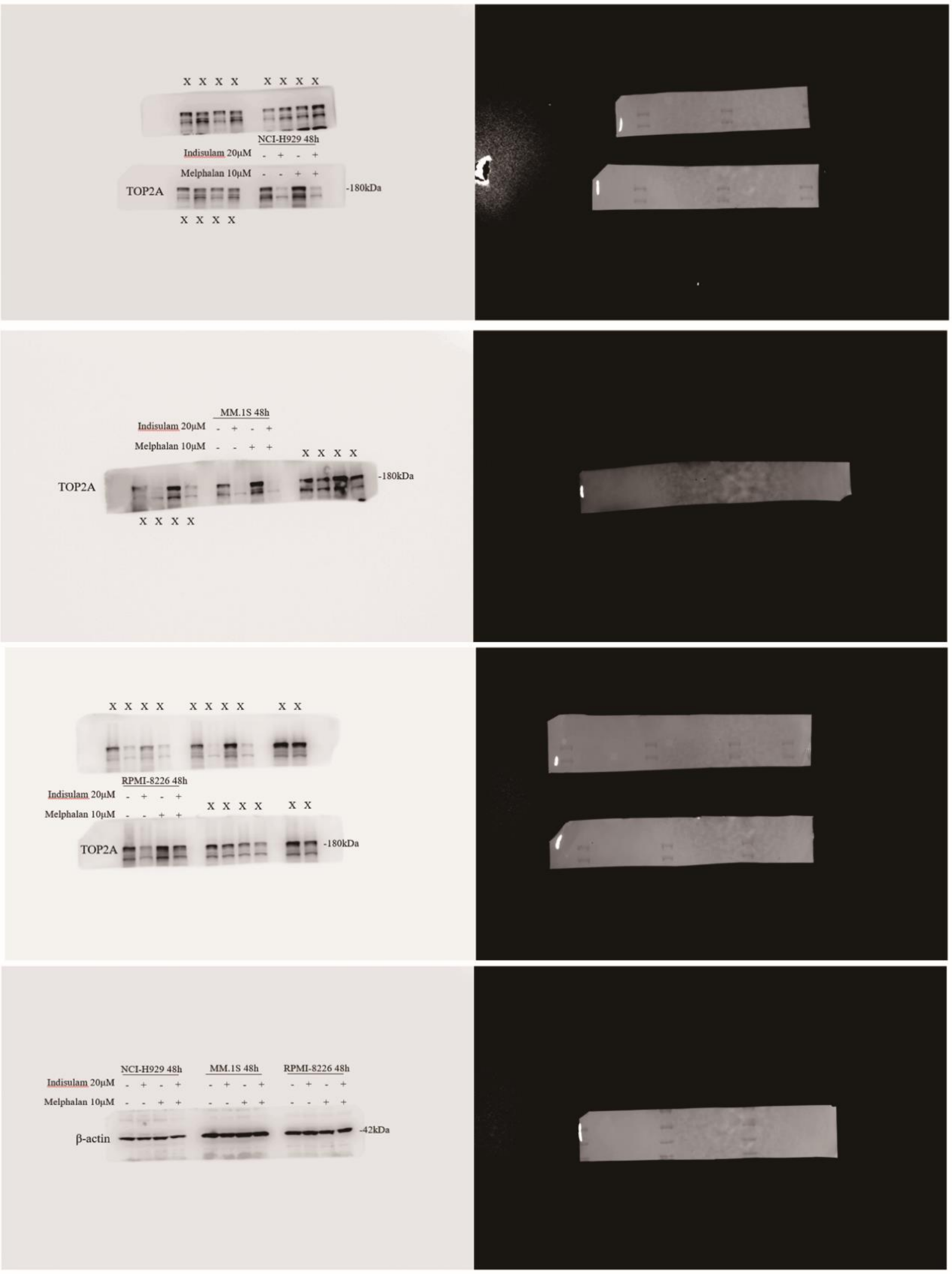
NCI-H929 48h
Indisulam 20μM
Melphalan 10μM
TOP2A
-180kDa
MM.1S 48h
Indisulam 20μM
Melphalan 10μM
TOP2A
-180kDa
RPMI-8226 48h
Indisulam 20μM
Melphalan 10μM
TOP2A
-180kDa
NCI-H929 48h
MM.1S 48h
RPMI-8226 48h
Indisulam 20μM
Melphalan 10μM
β-actin
-42kDa

Figure S3

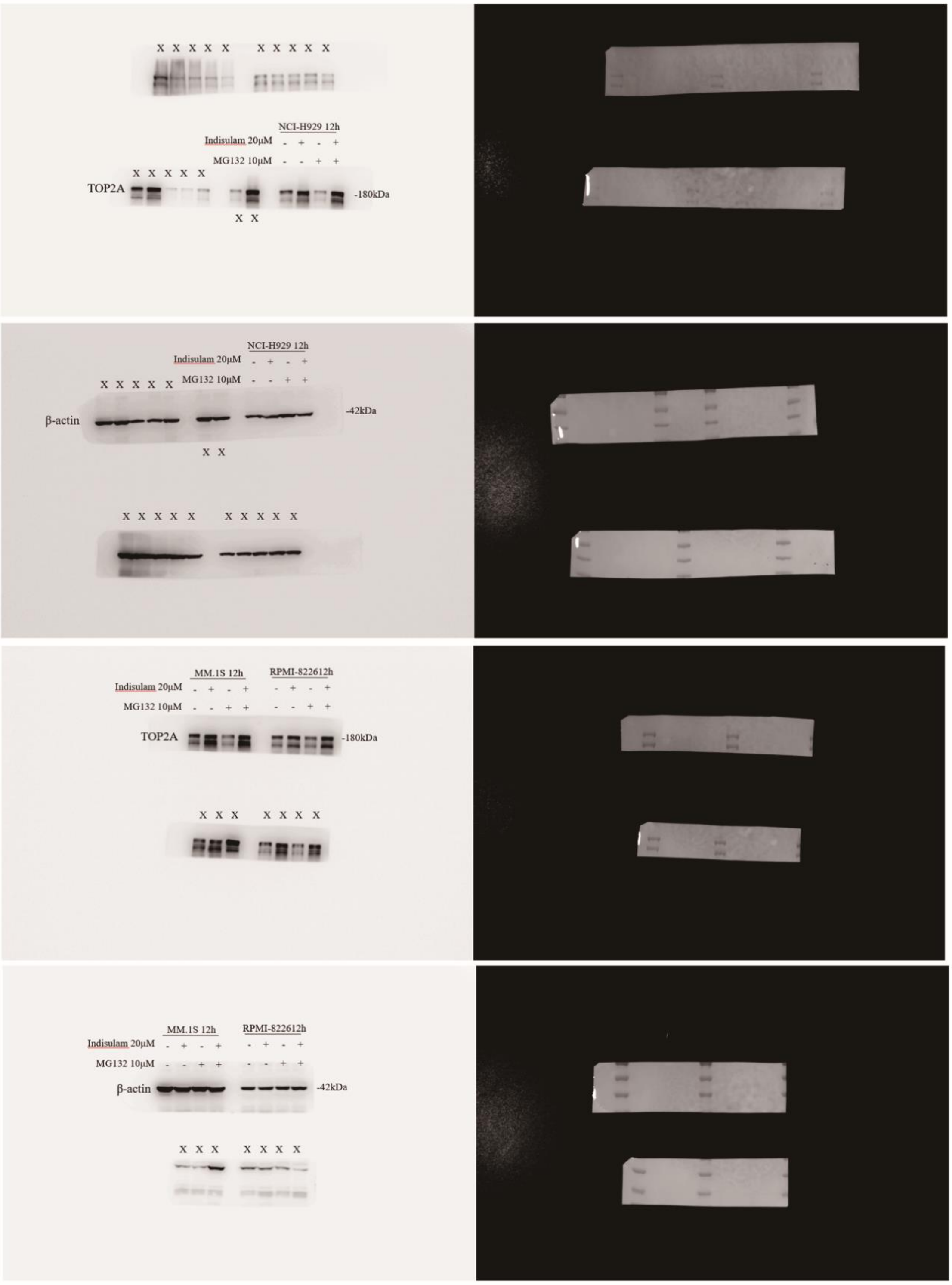
NCI-H929 12h
Indisulam 20μM
MG132 10μM
TOP2A
-180kDa
NCI-H929 12h
Indisulam 20μM
MG132 10μM
β-actin
-42kDa
MM.1S 12h
RPMI-822612h
Indisulam 20μM
MG132 10μM
TOP2A
-180kDa
MM.1S 12h
RPMI-822612h
Indisulam 20μM
MG132 10μM
β-actin
-42kDa

Figure 1f

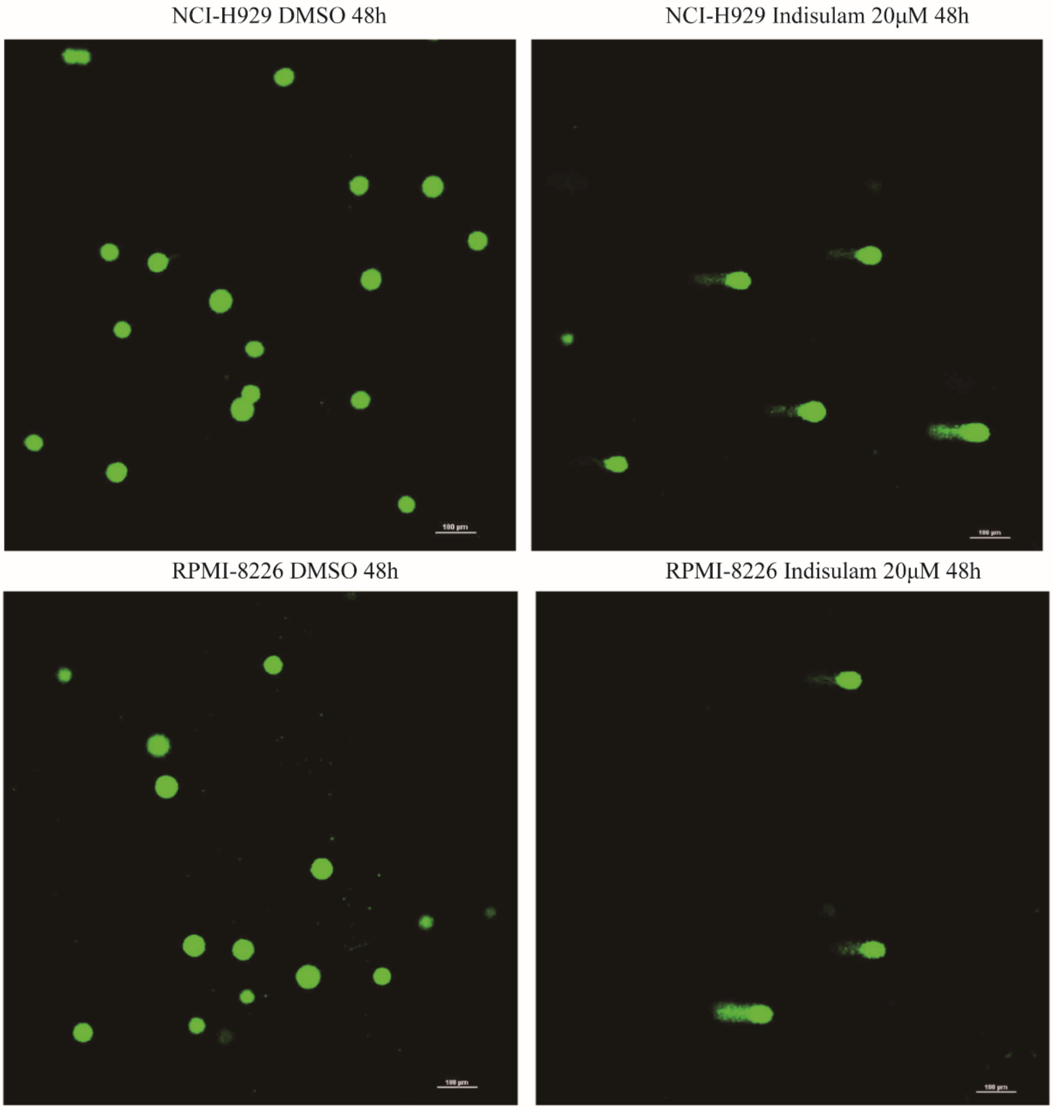
NCI-H929 DMSO 48h
NCI-H929 Indisulam 20μM 48h
RPMI-8226 DMSO 48h
RPMI-8226 Indisulam 20μM 48h

Figure 4d

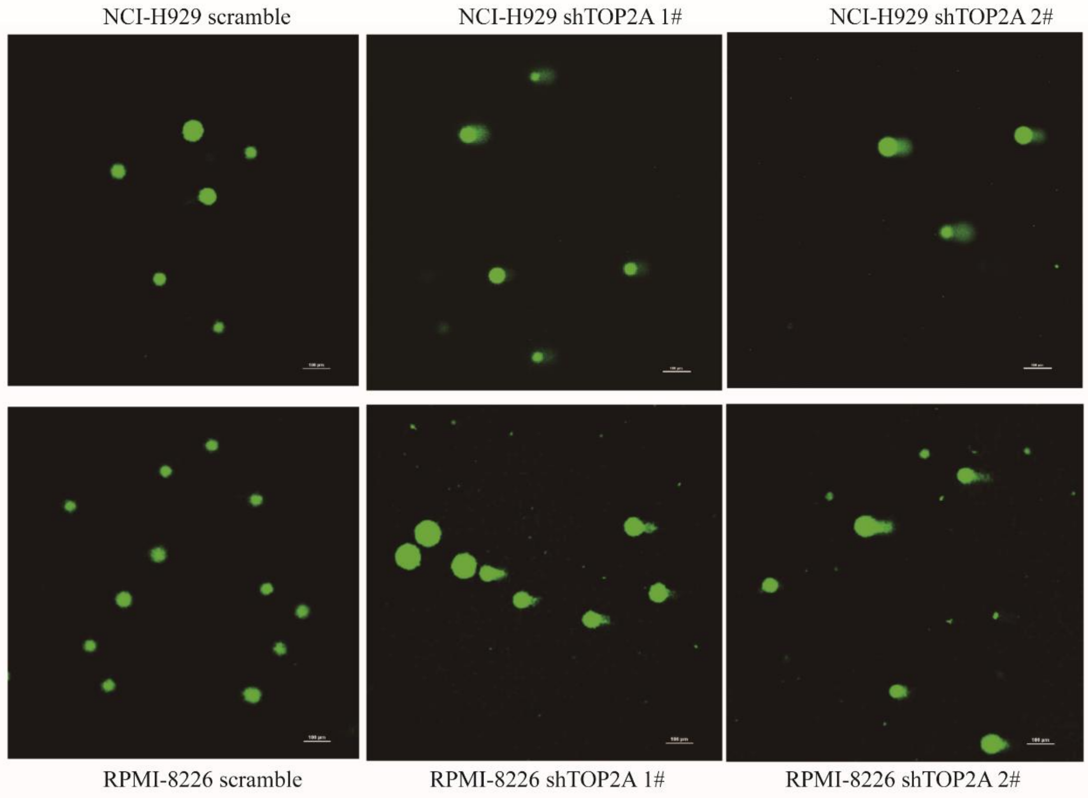

NCI-H929 scramble
NCI-H929 shTOP2A 1#
NCI-H929 shTOP2A 2#
RPMI-8226 scramble
RPMI-8226 shTOP2A 1#
RPMI-8226 shTOP2A 2#
